# Anisotropic Hydrogel Fibers for Soft Robotics: From Structural Engineering to Multi-Responsive Actuation

**DOI:** 10.3390/gels12080671

**Published:** 2026-07-27

**Authors:** Jian Zhang, Tianyu Wu, Ting Huang, Yang Zhang, Kai Hou, Guoyin Chen, Meifang Zhu

**Affiliations:** 1 State Key Laboratory of Advanced Fiber Materials, College of Materials Science and Engineering, Donghua University, Shanghai 201620, China; v1oletzz@163.com (J.Z.); 2250420@mail.dhu.edu.cn (T.W.); a15155972779@163.com (T.H.); houkai711@dhu.edu.cn (K.H.); zmf@dhu.edu.cn (M.Z.); 2 National Engineering Lab for Textile Fiber Materials & Processing Technology, Zhejiang Sci-Tech University, Hangzhou 310018, China; yzhang@zstu.edu.cn

**Keywords:** hydrogel fibers, soft robotics, anisotropic architectures, fabrication strategies, stimuli-responsive actuation, artificial muscles, functional integration

## Abstract

Hydrogel fibers provide a one-dimensional platform for constructing soft robotic materials that combine tissue-like compliance, high water content, structural anisotropy, and stimulus responsiveness. Compared with bulk hydrogels, their reduced radial dimensions shorten mass-transport pathways, while programmable fiber architectures convert otherwise isotropic swelling or contraction into directional deformation. This review summarizes the recent progress in anisotropic hydrogel fibers for soft robotics, with emphasis on the relationships among fabrication strategies, fiber architectures, actuation mechanisms, and robotic functions. Representative architectures, including Janus, bilayer, core–sheath, hollow, helically twisted, gradient, axially patterned, woven, and printed systems, are discussed in terms of their strain-conversion mechanisms, structural advantages, limitations, and suitable applications. Major fabrication approaches and stimulus-responsive mechanisms are further compared with respect to structural programmability, response kinetics, mechanical output, cyclic stability, scalability, and device integration. Particular attention is given to architecture selection, long-term environmental stability, interference from secondary stimuli, and the transition from laboratory demonstrations to practical soft robotic systems. Finally, key design principles and future directions are outlined for developing faster, more durable, manufacturable, and autonomous hydrogel-fiber-based soft robots.

## 1. Introduction

Over the past decade, soft robotics has developed into a distinct field beyond conventional rigid robotics, with a growing emphasis on compliant materials, continuum architectures, and distributed actuation. In natural organisms, structures such as tentacles and skeletal muscles are often built from highly oriented one-dimensional fibrous networks, including muscle fibers, collagen fibers, and nerve fibers [1,2,3]. In the context of “wet biomimetics,” hydrogels—soft materials composed of crosslinked polymer networks and water—have been widely explored as promising interfaces between soft robotic systems and biological tissues because of their high water content, low Young’s modulus, and multi-stimulus responsiveness [4,5,6].

Compared with other polymer-based actuator fibers, hydrogel fibers are distinctive not simply because they are compliant, but because their load-bearing polymer network remains permeated by a mobile aqueous phase. Dry fibers based on shape-memory polymers, liquid-crystal elastomers, carbon nanotube yarns, or dielectric elastomers generally provide higher output stress, faster cycling, and better dimensional stability in air [7]. Hydrogel fibers, by contrast, combine low modulus with ionic conductivity, molecular permeability, optical transparency, and direct coupling to water, salts, metabolites, enzymes, cells, and biological tissues. Their hydrated phase therefore functions simultaneously as a mechanical medium, a transport pathway, and a chemical or biological interface. This combination is difficult to reproduce in conventional dry polymer fibers and is particularly valuable for chemically driven artificial muscles, implantable interfaces, microfluidic robots, and systems that integrate actuation with optical, ionic, or molecular communication. The same aqueous phase also creates major disadvantages, including dehydration, freezing, osmotic drift, lower power density, and strong sensitivity to the surrounding medium. Hydrogel fibers are therefore most compelling when mass transport, biointegration, and multimodal function are as important as mechanical output [8].

One-dimensional hydrogel fibers offer an effective materials strategy for accelerating response kinetics while introducing structural anisotropy. Early studies showed that stimuli-responsive hydrogel systems, such as hydrolyzed polyacrylonitrile fibers and chitosan fibers, can undergo rapid swelling and contraction under chemical or pH stimuli. Their reduced radial dimensions markedly shorten the diffusion pathways of solvent molecules and ions, thereby accelerating dynamic response processes [9,10,11,12,13]. Compared with macroscale continuum soft robots, one-dimensional fibers have a higher specific surface area and can reduce radial diffusion lengths to the micrometer scale, substantially improving stimulus-response speed [14,15,16]. At the same time, their intrinsically one-dimensional geometry provides a versatile platform for structural engineering, including polymer-chain orientation, radial gradient construction, core–sheath design, multichannel arrangement, and interfacial heterostructuring.

This review treats the anisotropic hydrogel fiber as the fundamental mechanical and transport unit of a soft robot, rather than as a generic fiber-shaped sensor or a miniaturized bulk hydrogel. Particular attention is given to how cross-sectional and axial heterogeneity convert molecular-scale hydration, phase transition, ion redistribution, and field-induced forces into directional bending, twisting, contraction, pumping, and locomotion. The discussion links fabrication strategy, anisotropic architecture, nanocomposite transduction, actuation physics, environmental coupling, and system integration across scales, from single fibers to woven or printed robotic networks.

**Figure 1:**
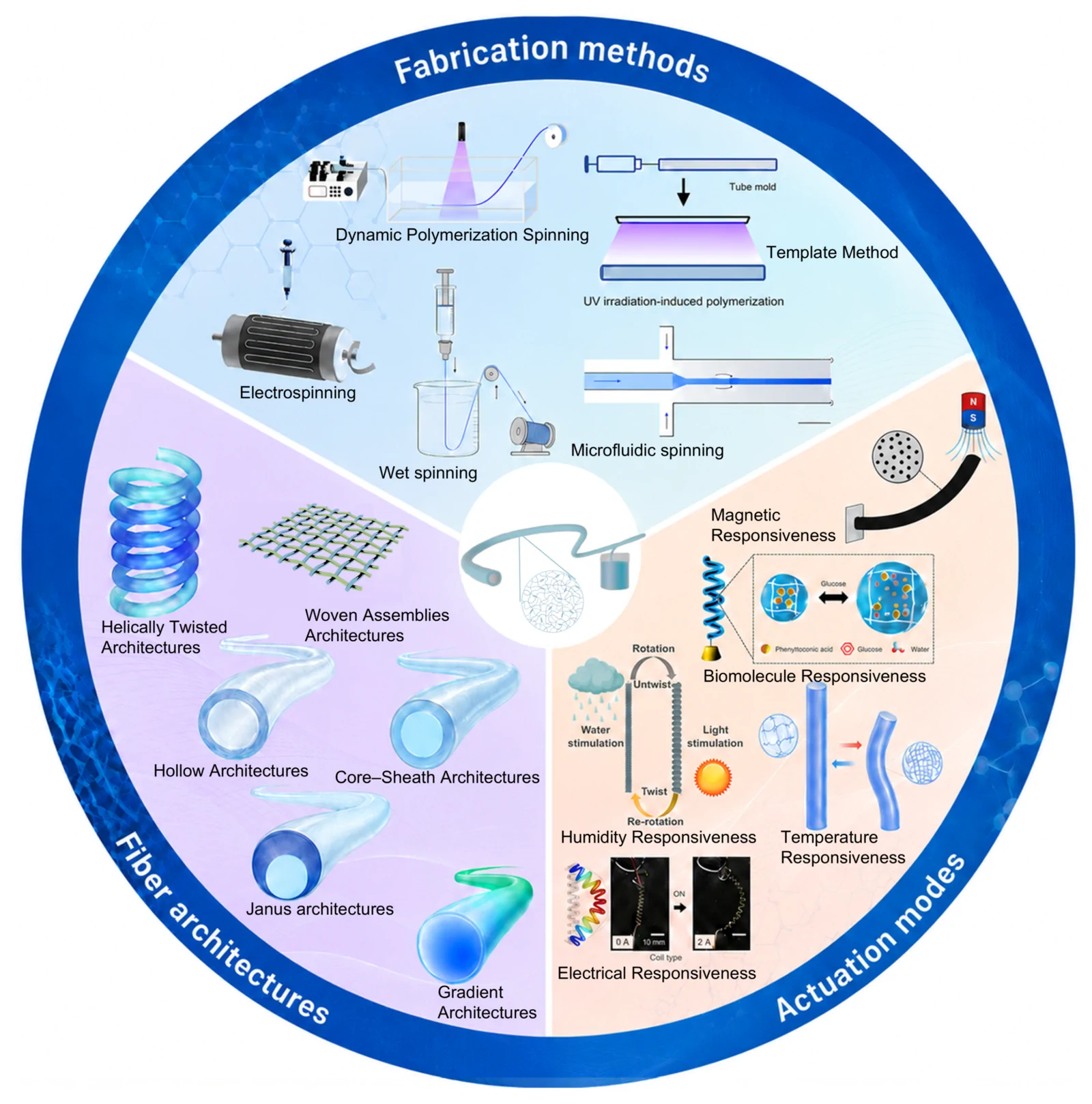
Schematic overview of hydrogel fibers for soft robotics: fabrication methods, fiber architectures, and actuation modes (humidity responsiveness, electrical responsiveness, and biomolecule responsiveness reprinted with permission from [17,18,19]).

Existing reviews have mainly organized hydrogel soft robots according to material type, external stimulus, or robotic function [1,2,4,7,15]. In this review, anisotropic fiber architecture is used as the main basis for comparison. We examine how cross-sectional and axial heterogeneity affect deformation direction, force transmission, response rate, and functional integration. The corresponding fabrication methods and actuation mechanisms are then compared in terms of structural precision, scalability, environmental stability, and device-level performance (Figure 1). This organization is intended to help readers select suitable fiber structures and processing routes for specific soft robotic tasks.

## 2. Anisotropic Architectures and Fabrication Strategies of Hydrogel Fibers

Anisotropic architecture and fabrication strategy are closely related but conceptually distinct. Architecture describes the spatial distribution of materials, interfaces, pores, molecular orientation, and functional domains within or among hydrogel fibers, whereas fabrication strategy determines how these structural features are introduced during precursor shaping, solidification, and continuous fiber formation. A single architecture may be produced by different fabrication methods, while one fabrication route may also generate several structural configurations. Distinguishing these two levels prevents mechanical function from being confused with process capability.

Directional actuation is governed primarily by how anisotropic architectures redistribute stimulus-induced strain. External stimuli first generate local free strain through swelling, contraction, phase transition, ion redistribution, or field-induced force. In a homogeneous and unconstrained fiber, this response mainly results in uniform dimensional change. In an anisotropic fiber, spatial variations in free strain, modulus, molecular orientation, or geometric constraint prevent uniform deformation and generate internal stress, bending moments, torque, or pressure. The resulting motion depends on the structural arrangement: transverse strain mismatch produces directional bending; radial confinement promotes axial contraction; circumferential or helical orientation converts volume change into torsion or contraction; lumen-volume variation enables pumping and fluid transport; and localized axial strain produces programmed folding or multistep deformation. Accordingly, the following section first discusses the major anisotropic architectures and their strain-conversion mechanisms, followed by a separate comparison of the fabrication strategies used to construct them.

### 2.1. Anisotropic Architectures and Strain-Conversion Principles

The actuation modes of hydrogel-fiber-based soft robots are largely governed by structural anisotropy. In fibers with homogeneous compositions and network architectures, external stimuli typically induce global swelling or contraction, producing relatively simple mechanical outputs [16,20,21,22,23,24]. By contrast, introducing heterogeneous architectures, such as Janus cross-sections, core–sheath structures, hollow channels, helical twists, gradient distributions, or axial patterning, allows stimulus-induced volumetric changes to be converted into directionally defined bending, elongation/contraction, twisting, coiling, and three-dimensional folding. These deformations arise from mechanisms such as cross-sectional strain mismatch [25], radial confinement [26], cavity-volume modulation [17], torsional strain release [27], and localized contraction [28]. Thus, the central design goal of hydrogel fibers is not only to enhance their intrinsic stimulus responsiveness, but also to control where, in which direction, and to what extent the response occurs through spatial structural programming. Through this strategy, molecular-scale hydration, dehydration, or chain-conformation transitions can be amplified into predictable and reproducible macroscopic motion [29].

#### 2.1.1. Janus and Bilayer Architectures

**Figure 2:**
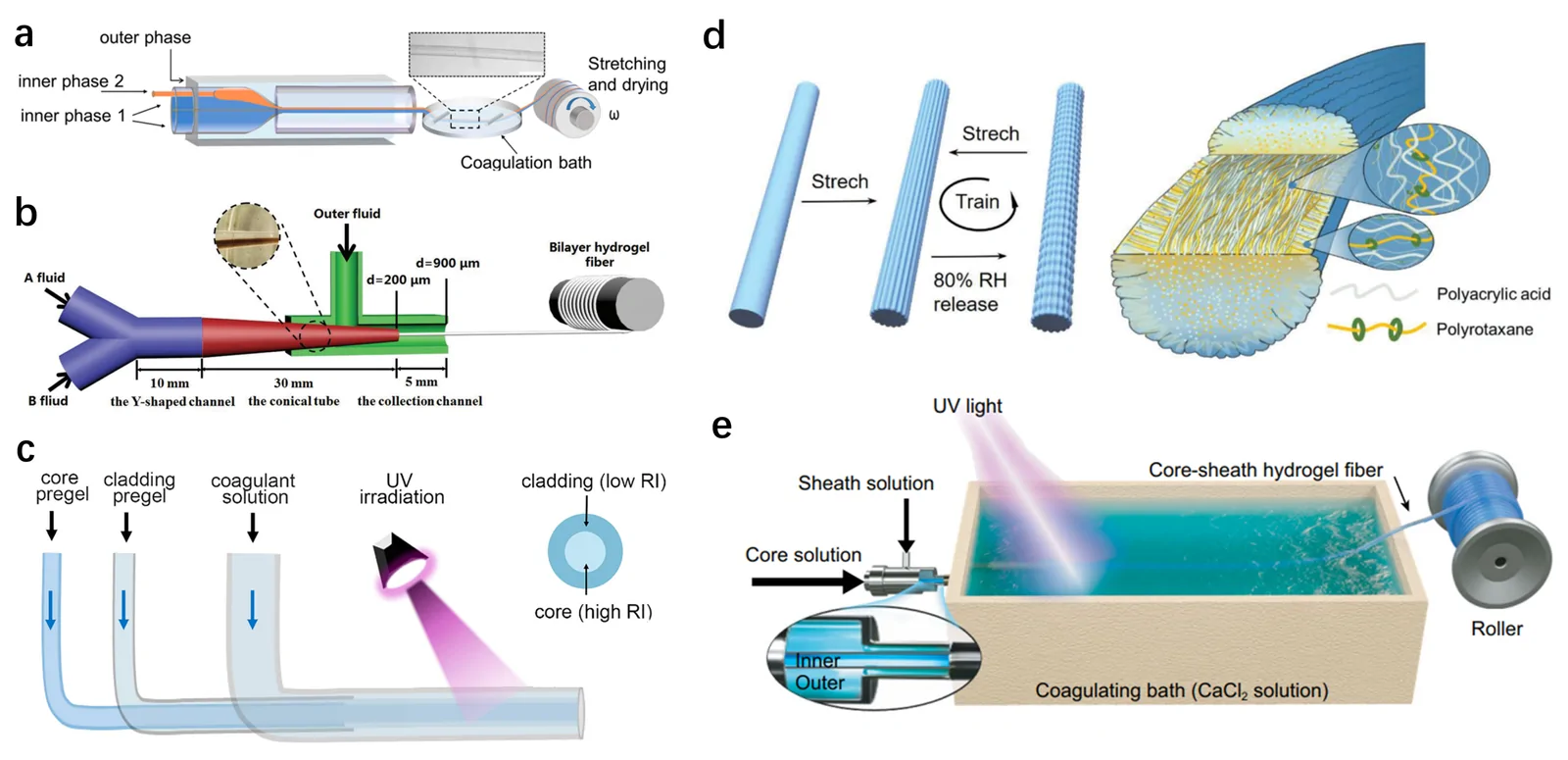
Fabrication of Janus, bilayer, and core–sheath hydrogel fiber architectures. (**a**) Microfluidic fabrication of Janus hydrogel microfibers and optical image of the resulting asymmetric fiber morphology (reprinted with permission from [20]). (**b**) Y-channel microfluidic spinning of bilayer hydrogel fibers with active and passive layers (reprinted with permission from [34]). (**c**) Coaxial microfluidic extrusion of dual-functional core–sheath hydrogel optical fibers for simultaneous light transmission and ionic conduction (reprinted with permission from [40]). (**d**) Stretch–release training of buckled sheath–core hydrogel fibers, showing the formation of an axially aligned core and radially buckled sheath (reprinted with permission from [39]). (**e**) Integrated dynamic wet spinning of core–sheath hydrogel optical fibers for tissue-compatible optical communication (reprinted with permission from [6]).

The central design principle of Janus fibers is to create stable swelling asymmetry across the fiber cross-section [20,25]. By introducing differences in material composition, crosslinking density, or functional components between the two sides of a fiber, stimulus-induced volumetric changes are converted from uniform elongation or contraction into an internal strain gradient across the cross-section [20,30]. This strain gradient can drive bending [31], coiling [32], or helical deformation of the fiber [33]. Hou et al. [20] fabricated Janus hydrogel fibers composed of sodium alginate and sodium alginate/silica composites using a θ-shaped channel microfluidic spinning strategy (Figure 2a). During dehydration, the two sides of the fiber shrank to different extents, causing the initially straight fiber to spontaneously coil into a tightly wound, tendril-like helical structure. This process transformed a two-dimensional linear fiber into a three-dimensional coiled configuration. By adjusting the silica content, the shrinkage constraint of the composite phase could be continuously tuned, allowing for precise control over the helix diameter and overall geometry of the coiled fiber. On the basis of this cross-sectional asymmetric design, Janus hydrogel fibers can undergo reversible coiling and uncoiling in response to ambient humidity changes. They can also generate localized deformation under local water stimulation or near-infrared photothermal stimulation, enabling functions such as micro-object lifting, long-distance pulling, and humidity-responsive switching. Overall, this architecture converts the intrinsic water absorption/desorption behavior of hydrogels into controllable three-dimensional deformation, providing a structural route for humidity-responsive hydrogel fiber actuators.

Bilayer hydrogel fibers operate on a principle similar to that of Janus structures, but place greater emphasis on the functional division between an active responsive layer and a passive constraining layer. Zhou et al. [34] continuously prepared hydrogel fibers with a well-defined bilayer architecture using a Y-shaped channel microfluidic spinning device (Figure 2b). One layer consisted of calcium alginate hydrogel, which served as the shape-maintaining and passive constraining component, while the other layer was composed of a PNIPAM/calcium alginate/GO semi-interpenetrating polymer network hydrogel, providing both thermoresponsive and photothermal responsiveness. The two precursor solutions formed stable laminar flows within the Y-shaped channel and were rapidly crosslinked in a Ca^2+^ coagulation bath, thereby creating a permanent asymmetric distribution of chemical composition across the fiber cross-section. By adjusting the flow rate of the spinning solutions and the winding speed, the fiber diameter could be tuned over the range of 80–500 μm. Because PNIPAM and GO were confined to the responsive layer, increasing temperature or near-infrared irradiation induced dehydration and contraction of this layer, whereas the calcium alginate layer remained relatively stable. The resulting interlayer strain mismatch drove directional bending of the fiber toward the responsive layer. This design integrates the thermally induced phase transition of PNIPAM, the photothermal conversion capability of GO, and the mechanical constraint of a bilayer cross-section within a single fiber, enabling external thermal or optical stimuli to be converted into directionally defined and reversible bending motion.

Janus and bilayer fibers are most efficient when one dominant bending axis is required, but the same transverse mismatch that generates high curvature also concentrates shear and residual stress at the interface. Initial bending angle alone is therefore an insufficient performance metric. Interfacial toughness, neutral-axis migration, swelling mismatch, and curvature retention under repeated wet–dry or thermal cycles should be reported before the architecture is selected for load-bearing operation.

#### 2.1.2. Core–Sheath Architectures

Core–sheath architectures provide a highly versatile structural format for hydrogel fibers [6]. Unlike Janus or bilayer fibers, which mainly rely on strain mismatch across an interfacial boundary [35], core–sheath fibers achieve functional compartmentalization through coaxial spatial organization. The core can be designed for mechanical reinforcement, electrical conduction, optical guidance, magnetic responsiveness, drug loading, or signal transmission [36,37,38], while the sheath can provide actuation, protection, interfacial adhesion, biocompatibility regulation, or environmental responsiveness [6,39]. Because of the continuous annular interface between the core and sheath, this architecture often offers greater structural stability than simple layered configurations during stress transfer, twisting, weaving, and long-term cyclic deformation [35,39]. Core–sheath hydrogel fibers are therefore particularly suitable for constructing multimodal fiber actuators, artificial muscles, and biomedical soft interfaces.

Singh et al. [40] fabricated conductive hydrogel optical fibers with a core–sheath structure through microfluidic coaxial extrusion. The fibers were based on an AAm/alginate double-network hydrogel. The core contained relatively high concentrations of AAm and alginate to form a high-refractive-index light-guiding region, while the sheath incorporated varying amounts of sodium acrylate to regulate its swelling degree and refractive index (Figure 2c). During fabrication, the core precursor, sheath precursor, and CaCl_2_ coagulation solution formed stable laminar flows within coaxial capillaries. Ca^2+^ first rapidly ionically crosslinked alginate to fix the core–sheath morphology, after which 365 nm ultraviolet irradiation initiated the covalent polymerization of AAm. This process produced a double-network structure consisting of an interpenetrating Ca–alginate ionic network and PAAm covalent network. After fiber formation, the hydrogel optical fibers were further immersed in 1.0 M NaCl to introduce Na^+^/Cl^−^ ion-conducting pathways, thereby endowing a single fiber with both optical transmission and ionic conductivity.

Sun et al. [39] proposed a distinct strategy for constructing buckled sheath–core hydrogel fibers through “Nano-Pulley Combing” and cyclic stretch–release training, improving mechanical performance and actuation capability. In their system, polyrotaxane (PR) was used as a sliding crosslinker, and polyacrylic acid hydrogel fibers were prepared by photopolymerization. During cyclic stretch–release training, the α-cyclodextrin rings in polyrotaxane acted like molecular “combs”, facilitating the axial alignment of polymer chains along the fiber direction. During the release stage, mechanical mismatch within the sheath induced chain buckling and folding, producing periodic buckled structures (Figure 2d). At the same time, the fiber core maintained strong axial molecular orientation, forming a heterogeneous architecture with an axially aligned core and a radially buckled sheath. This buckled sheath–core fiber combined high mechanical strength with humidity-responsive supercontraction, reaching a tensile strength of 1.61 GPa and a supercontraction stroke of 82% under humidity stimulation. By integrating molecular-scale sliding crosslinks, chain orientation, and macroscopic sheath buckling, this system endowed hydrogel fibers with high strength, high toughness, and humidity-driven actuation capability. It partially overcomes the conventional trade-off in hydrogels, which are typically soft but mechanically fragile and responsive but difficult to use under load. These features suggest potential applications in load-bearing hydrogel fiber actuators and cyclic actuation systems for hydrogel-fiber-based soft robotics.

Chen et al. [6] further extended the core–sheath concept to hydrogel optical fibers and biomedical soft interfaces by developing an integrated dynamic wet-spinning strategy. Using this approach, they continuously fabricated hydrogel optical fibers composed of a P(PEGDA-co-AAm) core and a calcium alginate sheath. The process coupled two gelation mechanisms within a single spinning procedure: ultraviolet-triggered free-radical polymerization formed a transparent covalent network in the core, while Ca^2+^-mediated ionic crosslinking rapidly solidified the sheath (Figure 2e). The synchronized operation of these two gelation processes enabled the continuous formation of hydrogel fibers with stable core–sheath cross-sections. By adjusting the monomer ratio in the core, the total monomer concentration, and the core/sheath extrusion-rate ratio, the core diameter, modulus, and optical transmission properties of the fibers could be flexibly tuned. In this system, the core provided low-loss optical transmission, while the sheath offered compliant protection and compatibility with tissue interfaces. The refractive-index contrast between the two regions established the optical guiding condition, allowing the fiber to achieve an optical attenuation as low as 0.18 dB cm^−1^ under 650 nm laser illumination. From a materials-design perspective, this work expands the role of core–sheath hydrogel fibers beyond the conventional “active layer/constraining layer” paradigm toward a functional division between an optical core and a biocompatible sheath. The core enables light transmission, the sheath provides soft protection and tissue-interface compatibility, and the overall hydrogel network helps reduce mechanical mismatch with soft tissues. Accordingly, core–sheath hydrogel fibers should not be viewed solely as actuator materials; they can also serve as flexible optical communication channels in soft robotics, bioelectronics, and neuromodulation systems.

The continuous annular interface makes core–sheath fibers suitable for protecting functional cores and separating multiple functions within one filament. However, a thick sheath may hinder mass transport, and radial swelling mismatch may damage the interface before whole-fiber failure becomes visible. Shell thickness should therefore be optimized by considering transport, load transfer, and protection together, and cyclic tests should assess core–sheath debonding in addition to tensile failure.

#### 2.1.3. Hollow Architectures

Hollow architectures represent an important structural format for the functional design of hydrogel fibers. Compared with solid hydrogel fibers, hollow fibers consist of a hydrogel wall and an internal lumen arranged radially. This geometry allows for stimulus-induced responses to extend beyond simple global swelling or contraction and to be further converted into lumen-volume regulation, fluid transport, micropumping, or intraluminal actuation [16]. On the one hand, the hollow channel shortens the diffusion distance of water, ions, and small molecules within the hydrogel wall; on the other hand, it provides an internal space for loading drugs, conductive liquids, magnetic particles, catalytic components, or cells [41]. Therefore, hollow hydrogel fibers should not be regarded merely as geometrically lightweight structures, but rather as an anisotropic design paradigm that couples mass transport, responsiveness, and actuation through a cross-sectional cavity. Microfluidic coaxial spinning is one of the most commonly used methods for fabricating hollow hydrogel fibers [42,43]. In this approach, the core fluid typically serves as a sacrificial template, while the shell fluid acts as the hydrogel precursor. After rapid crosslinking in an external coagulation bath, the core template is removed to obtain continuous hollow fibers. Compared with internal crosslinking strategies, external coagulation can avoid premature gelation inside the microfluidic channel and thus reduce clogging. Meanwhile, the outer diameter, inner diameter, and wall thickness of the fibers can be precisely controlled by adjusting the core/shell flow-rate ratio. This fabrication logic establishes a direct relationship between the geometric parameters of hollow structures and their functional responses.

**Figure 3:**
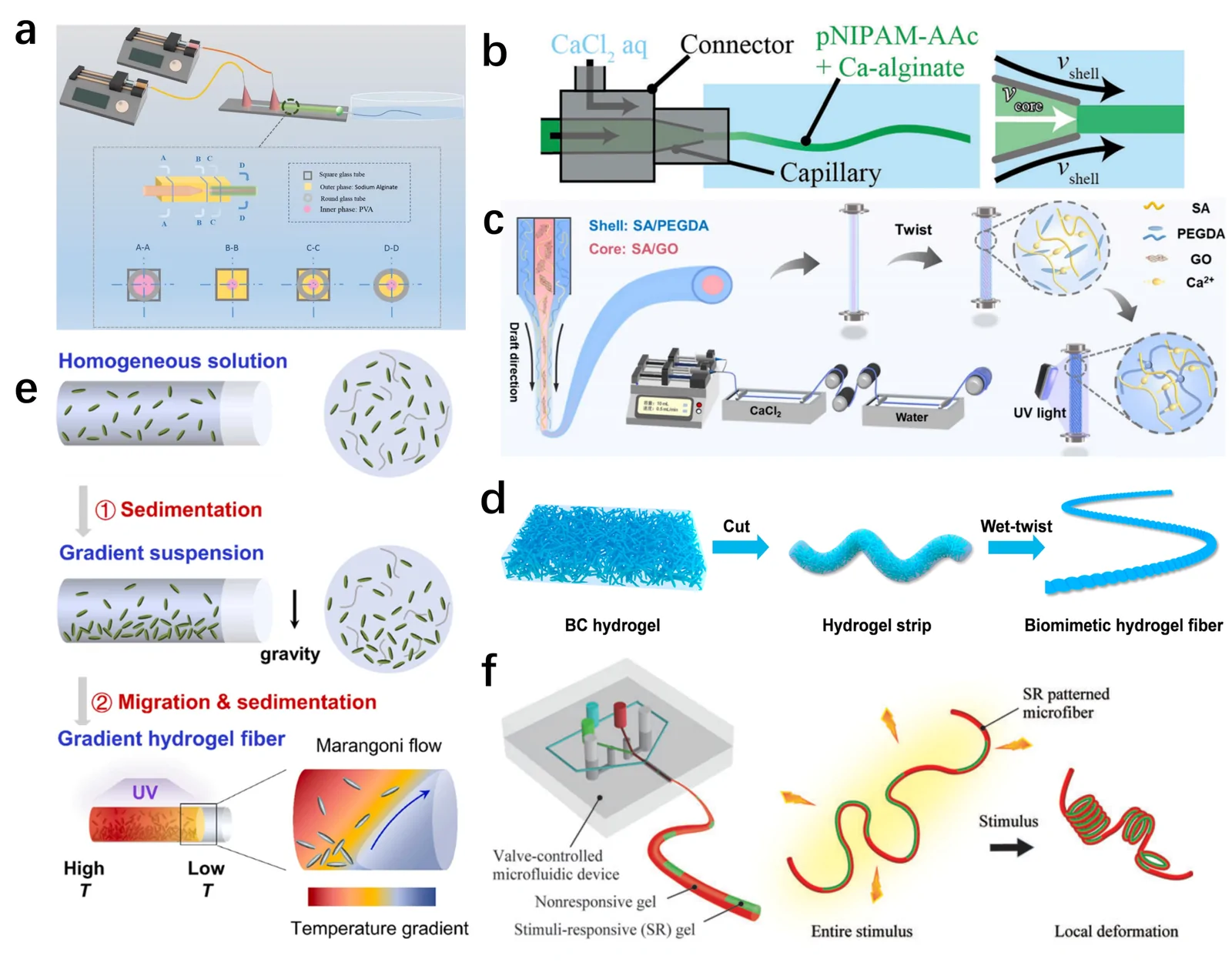
Fabrication of hollow, helically twisted, gradient, and axially patterned hydrogel fiber architectures. (**a**) Coaxial microfluidic fabrication of hollow hydrogel fibers using a sacrificial core template (reprinted with permission from [41]). (**b**) Stimuli-responsive hollow hydrogel microfibers with anisotropic shrinkage and lumen-volume modulation (reprinted with permission from [16]). (**c**) Preparation of shell–core helical alginate/PEGDA/graphene oxide hydrogel fibers through coaxial spinning, twisting, and UV crosslinking (reprinted with permission from [46]). (**d**) Bioinspired lotus-fiber-like helical bacterial cellulose hydrogel fibers formed by wet twisting and hydrogen-bond reconstruction (reprinted with permission from [49]). (**e**) Formation of dorsoventral gradient hydrogel fibers through TPE-CNC redistribution and Marangoni-flow-assisted self-organization (reprinted with permission from [22]). (**f**) Valve-controlled microfluidic fabrication of axially patterned hydrogel microfibers with programmable responsive and nonresponsive segments (reprinted with permission from [28]).

Wang et al. [41] prepared continuous hollow hydrogel fibers using a coaxial glass-capillary microfluidic device, in which sodium alginate solution was used as the shell phase and poly(vinyl alcohol) solution as the core template. The alginate shell was rapidly ionically crosslinked in a Ca^2+^ coagulation bath to form a calcium alginate hydrogel wall, followed by removal of the PVA core through washing (Figure 3a). The key feature of this system lies in fixing a transient coaxial fluid interface into a permanent hollow hydrogel cross-section through stable coaxial laminar flow and external ionic crosslinking. The core/shell flow-rate ratio determined the lumen size and hydrogel-wall thickness, thereby regulating the mass-transport pathway, mechanical compliance, and internal space available for functional-component loading. On this basis, different nanomaterials were incorporated into the alginate shell, allowing the same microfluidic platform to produce hollow fibers with distinct functions. CNT incorporation endowed the fibers with electrical conductivity and strain-sensing capability, yielding a conductivity of 15.8 S m^−1^ and an elongation at break of 342.9%. Fe_3_O_4_ incorporation introduced magnetic responsiveness, whereas MnO_2_ nanoparticles catalyzed oxygen-bubble generation in an H_2_O_2_ environment, driving self-propelled motion of the fibers with a maximum velocity of 615 μm s^−1^. In this case, the hollow lumen not only shortened the diffusion pathway but also provided internal space for bubble accumulation, release, and propulsion, allowing catalytic reactions to be translated into directional micromotion. The same hollow-fiber process was therefore used to produce electrically conductive, magnetically responsive, and catalytically propelled fibers by changing the nanofiller. The lumen also contributed to bubble accumulation and release in the MnO_2_-containing fibers, which supported directional self-propulsion.

Nakajima et al. [16] investigated stimulus-responsive actuation and microfluidic manipulation using hollow hydrogel fibers. They fabricated the fibers by microfluidic coaxial extrusion, using a shell solution composed of thermo-/pH-responsive poly(N-isopropylacrylamide-co-acrylic acid) [P(NIPAM-co-AAc)] and rapidly crosslinkable sodium alginate, with PVA serving as the sacrificial core template (Figure 3b). The resulting hollow fibers underwent large volumetric contraction when heated above 50 °C or when the pH was reduced below 2.5. Notably, the change in lumen volume was much greater than the change in the overall fiber diameter: when the fiber diameter decreased to 0.5 times its original value, the lumen volume decreased to only 0.1 times its initial value. Exploiting this feature, the authors injected a PVA solution containing fluorescent microbeads into the hollow channel and then triggered fiber contraction by lowering the pH, successfully pumping the microbeads out from the open end of the fiber. This demonstrated a stimulus-responsive micropumping function. The fibers also showed good reversibility during repeated contraction–swelling cycles, with the volume reversibly changing between 0.1 and 0.3 times its initial value. This work highlights the ability of hollow architectures to amplify stimulus-induced volume changes and provides a basis for their use in controlled drug release, microfluidic manipulation, and related soft robotic systems.

Hollow fibers are justified when the lumen contributes directly to pumping, delivery, fluidic conduction, catalytic propulsion, or loading of functional phases. Decreasing wall thickness accelerates exchange but simultaneously lowers collapse pressure and increases sensitivity to defects. Pressure–volume behavior, leakage, lumen patency, and wall fatigue are therefore more informative than external diameter change alone.

#### 2.1.4. Helically Twisted Architectures

Helical twisting is a key structural strategy for improving the mechanical performance of hydrogel fibers and enriching their actuation behaviors. Studies on fiber-based artificial muscles have shown that inserting twist into polymer fibers, and further converting them into coiled configurations, can efficiently translate volumetric changes or thermal contraction into large-amplitude torsional and axial contractile outputs [44]. In hydrogel fibers, twisted or coiled geometries can establish coupled axial–circumferential orientation within the fiber while storing releasable elastic energy through drying fixation, crosslinking-induced locking, or coil confinement. When external stimuli such as humidity, water, photothermal heating, or electroosmosis alter the hydration state and volume of the hydrogel network, the helical architecture guides strain release along predefined pathways, thereby generating reversible twisting, contraction, or elongation [3,17,45,46]. Thus, helical twisting is not merely a geometric modification, but a structural strategy for coordinating molecular orientation, energy storage, and deformation modes in hydrogel fibers [47]. Zhou et al. [48] systematically elucidated the working mechanisms of twisted fibers in artificial muscles, energy harvesting, and refrigeration. They pointed out that twist insertion can regulate mechanical properties by altering molecular orientation and packing within fibers, while volume changes can induce untwisting, coil contraction, or energy conversion. This mechanism is particularly relevant to hydrogel fibers because hydrogel networks are intrinsically prone to water absorption-induced swelling, dehydration-induced contraction, and ion-migration-mediated volumetric changes. Twisted or coiled architectures can convert these otherwise relatively uniform volume changes into amplified torsional or axial deformation. Accordingly, helical architectures provide an important geometric amplification mechanism that allows for swelling-induced volume changes to be converted into muscle-like torsional or axial actuation.

Wang et al. [46] designed a shell–core helical hydrogel fiber from the perspective of core–sheath functional differentiation and dual water/light responsiveness. The fiber was fabricated by coaxial wet spinning, using sodium alginate/poly(ethylene glycol) diacrylate (SA/PEGDA) hydrogel as the outer shell and sodium alginate/graphene oxide (SA/GO) hydrogel as the inner core. After spinning, the fiber was twisted and subsequently fixed by ultraviolet crosslinking to lock the helical configuration (Figure 3c). In this design, the SA/PEGDA shell formed a relatively stable double-network structure after twisting, which was responsible for water-absorption-induced swelling and preservation of helical morphology. The SA/GO core, by contrast, provided mechanical reinforcement and photothermal conversion through GO nanosheets. Through this core–sheath compartmentalization, water responsiveness and light responsiveness were assigned to different structural regions: the shell mainly governed moisture-induced swelling, whereas the core dominated photothermal dehydration and mechanical reinforcement. The twisting process served as the key step for deformation amplification. Under water stimulation, swelling of the shell triggered torsional release of the helical structure; under light irradiation, the GO-containing core absorbed optical energy and converted it into heat, promoting water removal and fiber recovery. Thus, the SA/PEGDA shell, SA/GO core, and helical geometry functioned as three coupled units corresponding to moisture-driven actuation, photothermal regulation, and torsional amplification, respectively. The resulting fibers could be further woven into humidity-responsive smart textiles, in which breathable pores opened under humid conditions and closed in the dry state, enabling potential applications in human thermal management. Compared with twisted fibers made from a single material, this shell–core helical architecture integrates multi-stimulus responsiveness, mechanical stability, and textile-level processability within one fiber platform through radial material compartmentalization and circumferential geometric twisting.

Guan et al. [49] developed lotus-fiber-inspired helical bacterial cellulose (BC) hydrogel fibers by mimicking the natural spiral configuration of lotus fibers. In this system, bacterial cellulose hydrogel membranes were first treated with alkali to remove bacterial residues and proteins, then cut into strips. A tangential twisting force was applied to the wet cellulose hydrogel strips by fixing one end and rotating the other end (Figure 3d). During wet twisting, hydrogen bonds between bacterial cellulose nanofibers were disrupted under external force, allowing slippage and plastic deformation of the three-dimensional nanofibrous network. After the external force was removed, hydrogen bonds re-formed, thereby fixing the lotus-fiber-like helical configuration.

Twisted and coiled fibers act as geometric amplifiers, but their output is highly sensitive to pretwist, pitch, chirality, fixation history, and hydration. Comparisons across studies should normalize torque, blocked force, stroke, and work by fiber mass or cross-sectional area and should report creep and torsional fatigue. A large free rotation without load does not necessarily indicate high useful work.

#### 2.1.5. Gradient and Axially Patterned Architectures

Beyond core–sheath, hollow, and helically twisted architectures, continuous cross-sectional gradients and axial patterning offer additional strategies for achieving programmable deformation in hydrogel fibers [22,28]. Cross-sectional gradients rely on gradual variations in composition, crosslinking density, or pore structure across the fiber, generating continuous anisotropy similar to the dorsoventral gradients observed in plant tendrils. Axial patterning, by contrast, arranges responsive and nonresponsive segments along the fiber length, giving one-dimensional fibers sequence-defined spatial responsiveness reminiscent of biological macromolecular encoding [23,50]. Together, these two strategies expand the design space of hydrogel fibers, shifting them from globally responsive materials toward programmable soft actuating units.

Inspired by the dorsoventral gradient structure of plant tendrils, Yu et al. [22] constructed a cross-sectional gradient network within a single continuous hydrogel fiber through Marangoni flow-induced self-organization. In this system, thermoresponsive PNIPAM hydrogel was used as the matrix, while tetraphenylethylene-labeled cellulose nanocrystals (TPE-CNCs) served as both functional nanofillers and fluorescent tracing units (Figure 3e). During fabrication, TPE-CNCs were first uniformly dispersed in the PNIPAM precursor solution and then allowed to partially sediment in a horizontal channel. Upon ultraviolet-initiated polymerization, the exothermic polymerization process further generated a temperature gradient within the system, inducing Marangoni flow that drove CNC migration toward the bottom side of the fiber. As a result, CNCs formed a gradient distribution across the fiber cross-section, with their concentration gradually increasing from the dorsal side to the ventral side. This cross-sectional gradient not only altered the local concentration of nanofillers, but also simultaneously modulated the local crosslinking density and pore structure of the hydrogel network. CNC-rich regions exhibited stronger physical constraints and a denser network, whereas CNC-poor regions remained relatively loose. Consequently, a continuous porous gradient ranging from nanopores to micropores was established across the fiber cross-section. This design converted an initially homogeneous thermoresponsive hydrogel into a structurally anisotropic fiber capable of programmable deformation, demonstrating how nanoscale filler redistribution can be used to encode macroscopic actuation behavior.

Takeuchi et al. [28] proposed an axial material-encoding strategy for programmable hydrogel microfibers. In this work, a valve-controlled microfluidic device was used to alternately introduce responsive and nonresponsive hydrogel precursor solutions during fiber fabrication. The responsive segments consisted of thermoresponsive P(NIPAM-co-AAc) and sodium alginate, whereas the nonresponsive segments were composed of pure sodium alginate. Both regions were rapidly ionically crosslinked in a Ca^2+^ coagulation bath and fixed as discrete functional segments within a continuous fiber (Figure 3f). By tuning the valve switching time, the length of the responsive segments could be controlled, with the minimum segment length reaching 2.3 mm, corresponding to an aspect ratio of approximately 17.2. The key feature of this axially patterned architecture is that it discretizes the responsive behavior along the fiber length. Upon heating, the P(NIPAM-co-AAc)-containing regions underwent dehydration-induced contraction, while the pure alginate regions remained relatively stable. The resulting difference in length change between adjacent regions generated localized bending and folding along the fiber. Because the position, length, and sequence of the responsive segments could be defined by the valve-switching program, the overall deformation of the fiber became spatially programmable. Fluorescence-labeling results further revealed that the fraction of the responsive region within the fiber cross-section did not reach a steady state instantaneously. Instead, it underwent a transition length of approximately 1.5 mm after the beginning of each patterned segment. This observation indicates that fluid switching, interfacial diffusion, and gelation kinetics during valve-controlled microfluidic processing collectively determine the spatial resolution of axial patterning. It also suggests that the precision of axially patterned hydrogel fibers is governed not only by the response time of the valve, but also by flow-field stability, precursor viscosity, diffusive mixing, and crosslinking rate. By integrating microfluidic valve control with rapid ionic crosslinking, this work enabled digital programming of axial functional domains in hydrogel fibers and provided a new paradigm for programmable deformation elements in soft robotic systems.

Continuous gradients avoid a discrete weak interface, whereas axial patterning enables sequence-defined local motion. Both strategies demand tighter process control than homogeneous or bilayer fibers. Gradient steepness, transition-zone length, batch-to-batch variation, and the stability of the encoded pattern after swelling should be quantified; otherwise, apparent programmability may reflect uncontrolled diffusion or gelation rather than reproducible structural encoding.

#### 2.1.6. Woven Assemblies and Printed Network Architectures

Woven assemblies and 3D-printed network structures offer effective routes for moving hydrogel fibers beyond single-filament actuation, enabling coordinated, multistep motions and macroscale task execution [51,52,53]. By organizing fibers through spatial arrangement, directional alignment, and multimateria integration, these architectures preserve the intrinsic merits of hydrogel fibers, such as large specific surface area and rapid responsiveness, while introducing diverse deformation modes and programmable locomotion capabilities into soft robotic systems [54].

**Figure 4:**
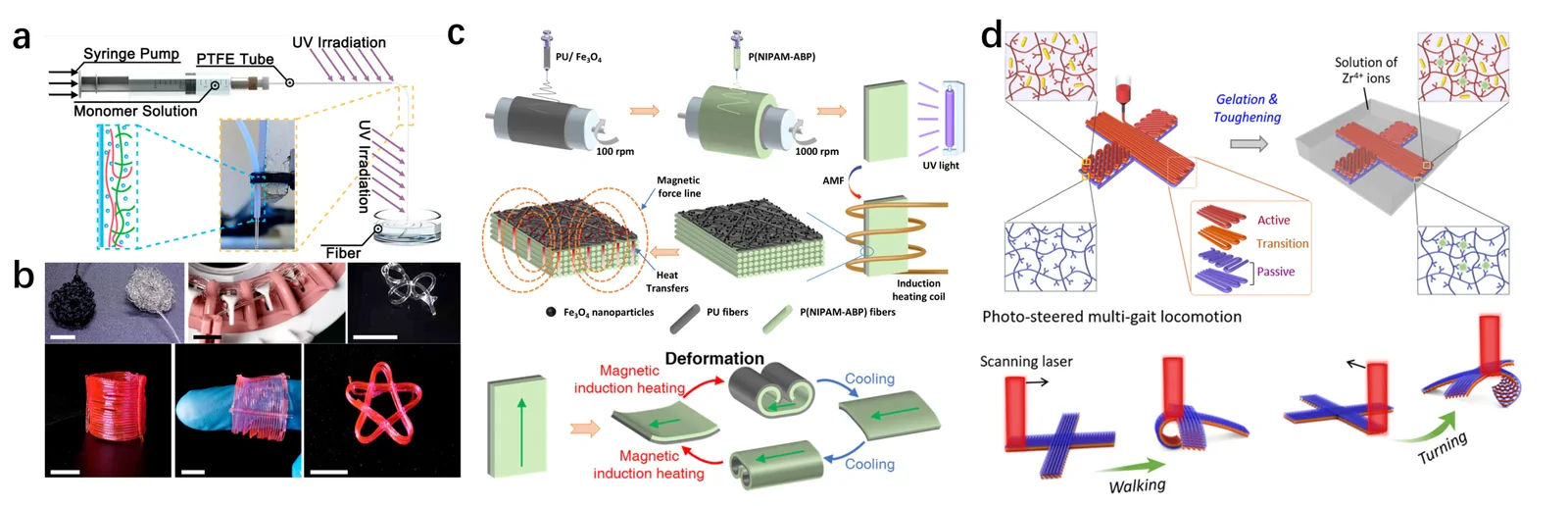
Assembly of hydrogel fibers into woven textiles and printed robotic networks. (**a**) Self-lubricated spinning apparatus for continuous fabrication of knittable PAMPS/PAAm hydrogel fibers (reprinted with permission from [51]). (**b**) Representative woven and knotted hydrogel-fiber architectures constructed from knittable hydrogel fibers; scale bar: 1 cm (reprinted with permission from [51]). (**c**) Preparation of PNIPAM/PU/Fe_3_O_4_ bilayer fibrous mats and their magnetothermal deformation under an alternating magnetic field (reprinted with permission from [52]). (**d**) Multimateria extrusion printing of tough hydrogel fiber networks for light-steered shape transformation and locomotion (reprinted with permission from [53]).

Duan et al. [51] developed a self-lubricated spinning strategy for the continuous fabrication of PAMPS/PAAm electroresponsive hydrogel fibers. In this method, linear PAMPS migrated toward the fiber surface during ultraviolet polymerization and formed a lubricating layer, which reduced adhesion between the fiber and the channel wall and enabled continuous demolding and long-length fiber production (Figure 4a). Subsequent solvent exchange with triethylene glycol increased the tensile strength of the fibers from 114 kPa to 5.6 MPa while maintaining their flexibility and weavability. The significance of this work lies not only in improving the mechanical properties of individual fibers, but also in giving hydrogel fibers processability comparable to that of conventional textile filaments. Through manual or machine-assisted weaving, the fibers could be assembled into various three-dimensional configurations, including flower-like structures, knots, tubular forms, pentagrams, and hollow cages (Figure 4b). For hydrogel-fiber-based soft robots, this “continuous spinning–solvent strengthening–woven assembly” route provides a cross-scale pathway from single electroresponsive fibers to macroscopic textile-like robotic architectures.

Shen et al. [52] fabricated PNIPAM/PU/Fe_3_O_4_ bilayer hydrogel fibrous mats by electrospinning. The mats consisted of a thermoresponsive PNIPAM fibrous layer and a magnetothermal PU/Fe_3_O_4_ fibrous layer, with Fe_3_O_4_ nanoparticles embedded in the PU fibers. Under an alternating magnetic field, these nanoparticles generated localized heat, which induced volumetric contraction of the PNIPAM layer. Because electrospinning produced pronounced alignment in the PNIPAM fibers, the mats contracted mainly along the fiber axis upon heating, with only limited transverse deformation. This anisotropic contraction resulted in in-plane directional shrinkage. By varying the cutting angle of the fibrous mats, the oriented shrinkage could be converted into different deformation modes, including curling, bending, and helical twisting (Figure 4c). This system demonstrates how fiber orientation, cutting direction, and macroscopic deformation can be coupled in fibrous membranes, enabling large-area hydrogel fiber assemblies to be actuated remotely and without contact under an alternating magnetic field.

Dong et al. [53] further extended hydrogel fiber assemblies to programmable printed networks using multimateria extrusion printing. In this system, nonresponsive P(AAc-co-AAm) hydrogel and photothermally responsive P(AAc-co-NIPAM) hydrogel were deposited through multiple nozzles along predefined paths to form multilayer fibrous grids, with network stability enhanced by Zr^4+^-mediated coordination crosslinking. After gold nanorods were incorporated into the responsive fibers, 808 nm near-infrared irradiation induced rapid photothermal heating, driving the local temperature above the lower critical solution temperature and triggering localized contraction. Because the orientation angle of the responsive fibers could be preprogrammed during printing, planar grids could transform into cylindrical or helical configurations depending on the fiber arrangement (Figure 4d). This work highlights the advantages of 3D printing for hydrogel fiber assemblies: material composition, fiber orientation, and spatial module distribution can be encoded synchronously within a single manufacturing process, enabling the structural programming of soft robotic motion from local responsive fibers to global robotic gait.

Woven and printed assemblies enable device-level locomotion, but they shift the dominant failure mode from the fiber body to knots, junctions, interlayer interfaces, and hydration nonuniformity. Printed networks offer superior geometric customization but lower throughput, whereas woven systems provide scalable assembly but limited local material resolution. Device-level output, connection durability, and performance after packaging should be evaluated in addition to single-fiber properties.

### 2.2. Fabrication Strategies for Hydrogel Fibers

#### 2.2.1. Wet Spinning and Continuous Photopolymerization

Wet spinning converts a flowing polymer or monomer solution into a filament through ionic crosslinking, solvent exchange, phase inversion, or coupled covalent polymerization. It is compatible with long fibers, post-drawing, twisting, coating, winding, and textile assembly, and is therefore one of the most practical routes for alginate-, chitosan-, polyacrylonitrile-, polyacrylamide-, and double-network hydrogel fibers [6,45,46,51]. Continuous in-tube photopolymerization and self-lubricated spinning further reduce adhesion to the forming channel and enable direct collection of meter-scale fibers. The main limitations are radial coagulation gradients, solvent and salt recovery, oxygen inhibition, light attenuation in particle-rich formulations, and a narrower ability to program abrupt axial or multichannel features.

#### 2.2.2. Microfluidic Spinning

Microfluidic spinning stabilizes multiple precursor streams as laminar co-flows and fixes them by rapid ionic, photochemical, or interfacial crosslinking. Y-shaped, theta-shaped, coaxial, multilayer, and valve-controlled channels can continuously generate Janus, bilayer, core–sheath, hollow, and axially patterned fibers with micrometer-scale control over compartment dimensions [16,20,28,34,41]. Its principal advantage is architectural precision rather than throughput. Channel clogging, precursor-viscosity mismatch, diffusive blurring between streams, pressure fluctuations, and sensitivity to gelation kinetics currently limit scale-up; parallelization and in-line dimensional monitoring are required before laboratory precision can be translated into robust production.

#### 2.2.3. Electrospinning, Template Methods, and Multimaterial Extrusion Printing

Electrospinning is efficient for aligned nanofibrous mats, porous bilayers, and large-area cilia-like assemblies, but it is less suitable for isolated, fully hydrated, load-bearing fibers with controlled lumens [52,55,56]. Template methods offer low equipment complexity and straightforward control of solid-fiber diameter, yet demolding friction and batchwise operation restrict length and structural complexity [57]. Multimaterial extrusion printing directly encodes material identity, fiber orientation, and network topology at the device scale [53]. Its advantages in customization and integration are balanced by slower production, stringent ink-rheology requirements, interlayer adhesion defects, and dehydration during long print cycles. These method-specific trade-offs and their preferred roles are summarized in Table 1.

### 2.3. Comparative Selection of Fiber Architectures

No fiber architecture is universally superior. Selection depends on the required deformation mode, number of integrated functions, transport requirements, cyclic load, and manufacturing tolerance. Janus and bilayer fibers maximize transverse strain mismatch and are efficient for planar bending; core–sheath fibers trade fabrication complexity for functional compartmentalization and interface continuity; hollow fibers provide rapid exchange and lumen actuation at the cost of collapse resistance; helical fibers amplify modest material strain into torsion or contraction but are sensitive to twist geometry and fatigue; gradient and axially patterned fibers offer continuous or sequence-programmed motion but require tighter process control; and woven or printed networks enable system-level tasks while introducing connection, hydration, and defect-management challenges. These distinctions are summarized in Table 2.

From a design standpoint, the preferred architecture is the simplest structure that generates the required degree of freedom while preserving load transfer, cyclic stability, and manufacturability. Additional structural complexity is justified only when it provides a measurable gain in motion programmability, multifunctionality, transport capability, or device-level integration.

### 2.4. Nanocomposite Functionalization of Hydrogel Fibers

Nanofillers can reinforce hydrogel fibers or introduce electrical, magnetic, photothermal, catalytic, and imaging functions. Their effect depends strongly on their dispersion, orientation, and connectivity within the fiber. Discrete nanoparticles can provide localized magnetic, catalytic, or photothermal activity; nanotubes and nanofibers can form conductive or load-bearing pathways; and nanosheets can increase interfacial stress transfer and light absorption. These benefits must be balanced against aggregation, particle leakage, optical attenuation, and restricted water transport.

Graphene oxide and reduced graphene oxide are the most widely used carbon nanomaterials in the systems surveyed here. They reinforce alginate or PNIPAM networks, absorb light for photothermal dehydration, and assist the formation of helical or gradient structures [17,22,45,46,58]. Carbon nanotubes can form conductive sheaths or percolated pathways that improve electrical response and constrain radial swelling [21,41,59]. These benefits are offset by aggregation, nonuniform percolation, optical attenuation, and possible particle release. A high filler fraction may improve conductivity or heating while reducing water transport and actuation strain; dispersion quality and interfacial load transfer must therefore be quantified alongside bulk filler content.

Cellulose nanocrystals, bacterial cellulose nanofibers, magnetic nanoparticles, gold nanorods, and MnO_2_ catalytic nanoparticles provide complementary functions. Cellulose nanomaterials introduce orientation, hydrogen-bonded reinforcement, and gradient formation [22,30,49]; Fe_3_O_4_ enables magnetic force, torque, imaging, or magnetothermal heating [30,41,52]; gold nanorods provide efficient near-infrared heating in printed networks [53]; and MnO_2_ acts as a nanozyme-like catalyst that converts H_2_O_2_ into oxygen bubbles for self-propulsion [41]. For these systems, aggregation, sedimentation during spinning, interfacial debonding, leaching, local overheating, and changes in refractive index or swelling must be treated as coupled design constraints.

MXenes are an emerging two-dimensional platform because their high conductivity, hydrophilic surfaces, and photothermal response can couple sensing, Joule heating, and optical actuation. Ti_3_C_2_T_x_ nanosheets have been shown to accelerate hydrogel gelation and support extrusion of long, stretchable, self-healing hydrogel fibers [60], but MXene-enabled hydrogel-fiber actuators remain less developed than GO- or CNT-based systems. Oxidation in water and air, conductivity drift, nanosheet restacking, and uncertain long-term biological fate are substantial barriers. Future nanocomposite fibers should therefore prioritize anisotropic or compartmentalized filler placement, stable polymer–nanofiller interfaces, and retention of function after cyclic swelling, rather than simply maximizing nanoparticle loading.

### 2.5. Manufacturing Scalability and Device Integration

Wet spinning and continuous photopolymerization are currently well suited to long-fiber production, winding, drawing, twisting, and textile post-processing [6,51,61]. Microfluidic spinning provides finer control over Janus, core–sheath, hollow, and patterned cross-sections, but its throughput is limited by channel clogging, viscosity mismatch, pressure fluctuations, and gelation kinetics [16,20,28,41]. Electrospinning is useful for aligned mats and bilayer membranes, whereas multimaterial extrusion printing allows spatial control of composition and network geometry. The appropriate method therefore depends on whether the priority is fiber length, cross-sectional precision, sheet-scale assembly, or direct device fabrication [53].

For long yarn actuators, wet spinning can be combined with twisting, coating, and weaving. Microfluidics is more appropriate when precise compartmentalization is required, while electrospinning is suited to fibrous membranes and cilia-like arrays. Multimaterial printing is advantageous for customized networks, although its production rate and material window remain limited. Future scale-up studies should report run length, production rate, diameter variation, defect rate, solvent recovery, and performance after assembly.

## 3. Environmental Responsiveness and Actuation Mechanisms of Hydrogel-Fiber-Based Soft Robots

Soft robotic actuation generally relies on converting external energy inputs into mechanical stresses within polymer networks [53,62]. The mesoscopic geometry of one-dimensional fibers can shorten diffusion pathways, introduce directional architectures, and provide greater structural freedom for multi-field actuation [52,63]. Conventional volumetric phase-transition mechanisms, such as the smectic mesophase transition of liquid-crystalline hydrogels near physiological temperature, illustrate the polymer-physics basis of thermoresponsive actuation. In these systems, phase transitions can drive water redistribution between amorphous and ordered domains, leading to macroscopic contraction [64]. However, because such mechanisms typically depend on solvent migration within the hydrogel network, their response kinetics are inherently limited by diffusion processes [65,66]. To achieve faster, remote, and non-contact actuation, increasing attention has therefore turned to physical-field-driven mechanisms, including electrical, magnetic, thermal, and humidity-based stimulation (Table 3).

**Table 3:** Summary of major actuation modes for anisotropic hydrogel fibers.

Stimulus Category	Main Mechanism	Typical Motion	Main Characteristics	Ref.
Temperature-responsive	Thermally induced phase transition, dehydration, and network contraction	Contraction, bending, folding, and shape recovery	Reversible and structurally programmable, but generally limited by heat and water diffusion	[16,22,24,28,34,55,58,61,67,68,69]
Water/Humidity-responsive	Water absorption, dehydration, swelling mismatch, and release of stored torsional strain	Torsion, rotation, contraction, extension, and bending	Simple stimulation and large deformation, but strongly affected by ambient humidity and drying conditions	[17,21,30,39,45,46,47,57,70,71]
Light/Photothermal-responsive	Photothermal conversion induces local heating, dehydration, or phase transition	Bending, curling, walking, steering, and cargo transport	Remote and spatially selective control, but limited by light penetration and uneven heating	[31,53]
Electrical/Electrochemical/Electromagnetic	Ion migration, electroosmosis, electrochemical reactions, conductive-network changes, or Lorentz force	Bending, vibration, contraction, extension, and gripping	Rapid and compatible with electronic control, but may require electrodes, conductive media, or external magnetic fields	[3,9,10,12,18,44,51,56,59,72,73]
Magnetic-responsive	Magnetic force, torque, particle alignment, or magnetothermal conversion	Steering, rolling, jumping, swinging, and targeted locomotion	Wireless and suitable for confined or biological environments, but dependent on particle loading and magnetic-field configuration	[37,41,42]
pH/Ion/Biomolecule-responsive	Ionization, coordination, osmotic-pressure changes, or specific molecular recognition	Swelling, contraction, rotation, and axial actuation	Suitable for chemical and physiological environments, but sensitive to ionic strength, buffer composition, and competing species	[19,74,75,76,77,78]

### 3.1. Humidity Responsiveness

Water/humidity responsiveness represents a common and important actuation mode of hydrogel fibers, and it originates from the reversible volumetric changes of hydrophilic polymer networks during water uptake and water loss [13,70,79]. Hydrophilic groups such as carboxyl, hydroxyl, amide, and sulfonate moieties can form hydration shells with water molecules, causing the fibers to swell when exposed to elevated humidity or liquid water. Under dry conditions or photothermal heating, water is removed and the network contracts [17]. In homogeneous solid fibers, this process is typically manifested only as axial or radial dimensional change [80]. However, when hollow lumens, helical twists, prestrained configurations, or hierarchically twisted architectures are introduced, water exchange can be further translated into more complex actuation modes, including torsion, extension/contraction, coiling, contraction recovery, and micropumping.

**Figure 5:**
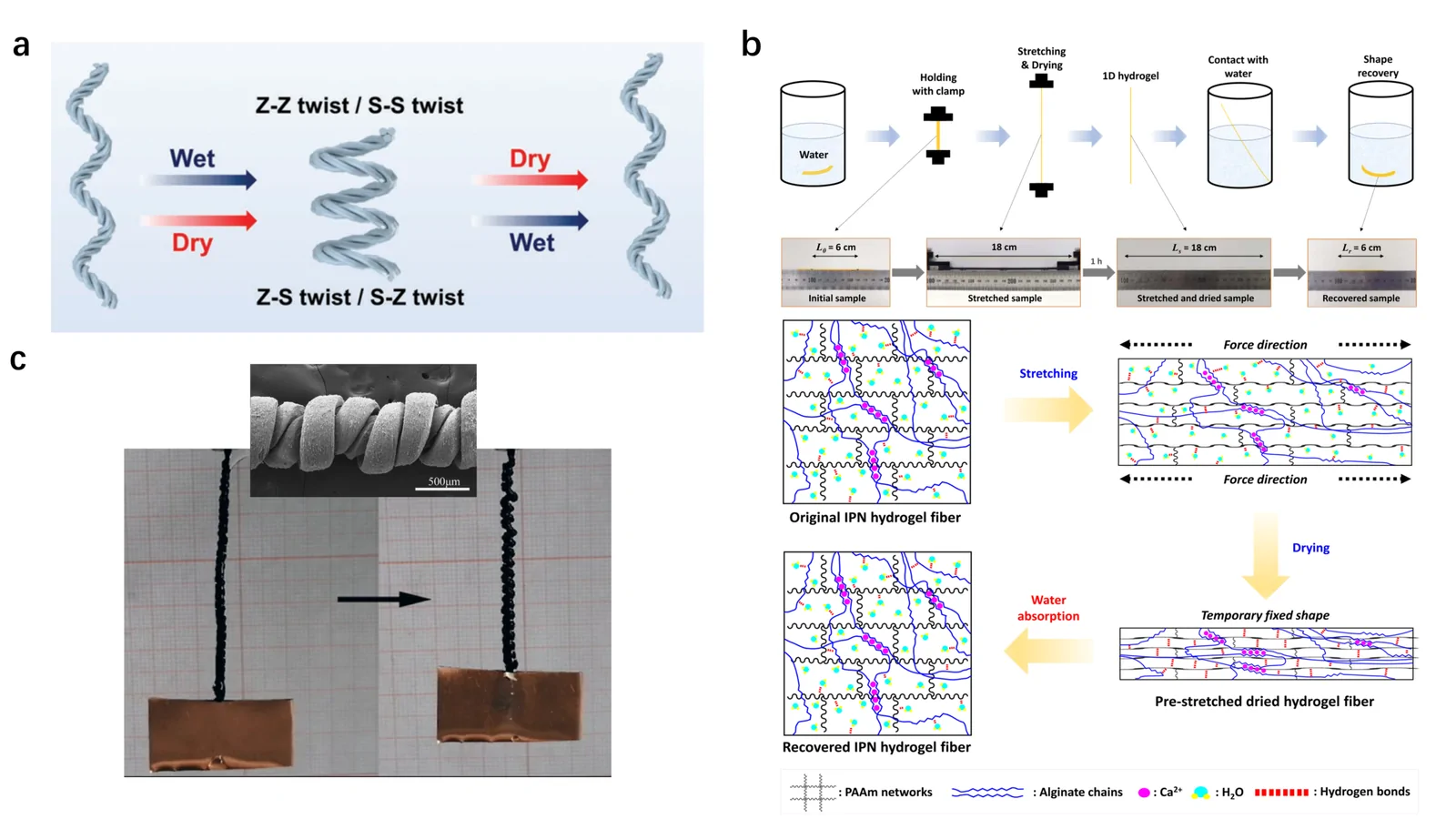
Water- and humidity-driven actuation of hydrogel fibers through swelling, shape memory, and torsional release. (**a**) Humidity-responsive torsional and axial actuation of coiled RGO-loaded hollow hydrogel fibers, showing contraction in homochiral Z–Z/S–S assemblies and extension in heterochiral Z–S/S–Z assemblies (reprinted with permission from [17]). (**b**) Water-triggered shape recovery of prestrained interpenetrating-network hydrogel fibers through stretching, drying fixation, and immersion in deionized water (reprinted with permission from [57]). (**c**) Water-responsive untwisting actuation of helically structured GO/alginate hydrogel fibers driven by swelling-induced release of stored torsional strain (reprinted with permission from [45]).

The reduced-graphene-oxide-loaded hollow hydrogel fiber (RGO@HHF) developed by Liu et al. [17] illustrates how hollow architecture and helical twisting can synergistically amplify water-responsive actuation. The fiber was first fabricated as a hollow hydrogel filament by wet spinning and was then converted into a doubly twisted structure through twisting, folding, plying, and rewinding (Figure 5a). The hollow lumen reduced the effective water-uptake volume and shortened the water-transport pathway, enabling the hydrogel wall to swell rapidly under humidity stimulation. Meanwhile, the helical twist transformed radial swelling into untwisting rotation. The torsional angle of a single-twisted RGO@HHF reached 3168° cm^−1^, with a rotational speed of up to 132 rpm. After construction of a doubly twisted structure, the axial output could be further regulated by chirality: homochiral assemblies exhibited contraction, whereas heterochiral assemblies showed extension. The role of RGO was mainly associated with photothermal regulation, as its strong solar absorption enabled rapid dehydration of the fiber under light irradiation, thereby driving reverse recovery. This system integrated “water absorption–hydrogel-wall swelling–helical untwisting–photothermal dehydration recovery” into a reversible actuation cycle, demonstrating that hollow and twisted architectures can increase the response rate and angular output of humidity-responsive hydrogel fibers.

Kim et al. [57] employed a water-triggered shape-memory mechanism to convert the water-uptake process into axial contraction recovery. Their PAAm/calcium alginate interpenetrating-network hydrogel fibers were prepared by a templating method, in which the PAAm network provided flexibility and elastic recovery, while the calcium alginate network contributed energy dissipation and shape stabilization. Wet fibers were first stretched and then dried at room temperature, during which interchain hydrogen bonding and vitrification fixed the prestrained configuration. Upon re-exposure to water, water molecules disrupted the interchain interactions in the dry state, allowing the network to transition from a glassy state back to a rubbery state, release the stored elastic stress, and contract to nearly its original length within approximately 1 min. The key actuation mechanism in this system was therefore not continuous swelling, but a shape-memory process involving “prestraining–drying fixation–water-triggered release” (Figure 5b). Compared with bending actuation that relies on real-time humidity gradients, this strategy allows mechanical pretreatment to be encoded into a temporary dry-state shape and then rapidly converted into contraction, bending, or lifting motions in aqueous environments.

An et al. [45] constructed a hierarchically arranged helical structure with anisotropic organization in GO/alginate hydrogel fibers through wet spinning combined with confined drying. In this approach, GO/alginate hydrogel fibers were first prepared by Ca^2+^ crosslinking, and then stepwise twisted and dried under fixed-end conditions so that primary and secondary helical structures were sequentially introduced into the fibers. GO nanosheets reinforced the fiber network and helped stabilize the oriented structure, whereas the alginate network provided water-responsive swelling capability. When the dry helical fibers were exposed to water, the hydrogel network swelled and released the torsional strain stored during twisting, thereby generating untwisting rotation. The secondary helical structure further amplified the volume-change-induced motion, enabling a single fiber to rotate by more than 200° and to drive a load approximately 20 times its own weight (Figure 5c). This system indicates that humidity-responsive actuation depends not only on the hydrophilicity of the material itself, but can also be significantly enhanced by post-processing routes such as “wet spinning–constrained twisting–drying fixation”, which pre-store releasable hierarchical torsional energy within the fiber.

Humidity-driven fibers are chemically simple and can produce large strokes, but their baseline depends on relative humidity, airflow, temperature, exposed surface area, and previous drying history. They are particularly suitable for environmental energy harvesting and adaptive textiles; precision robotic control requires reference sensors, controlled vapor delivery, or packaging that stabilizes the water chemical potential.

### 3.2. Temperature Responsiveness

Temperature-responsive hydrogel fibers usually rely on hydration/dehydration transitions of thermoresponsive polymer chains, among which poly(N-isopropylacrylamide) (PNIPAM) and its copolymers are the most representative systems [16]. Below the lower critical solution temperature (LCST), PNIPAM forms stable hydration shells with water molecules, and the hydrogel network remains in a swollen state. When the temperature rises above the LCST, hydrophobic interactions become dominant, leading to polymer-chain collapse, water expulsion, and network contraction [81,82]. For hydrogel fibers, the key issue in temperature responsiveness is not only the phase transition itself, but also how chain orientation, fiber-bundle assembly, or axial patterning can convert an otherwise nearly uniform volumetric contraction into axial contraction, local curling, or three-dimensional folding [28,61].

**Figure 6:**
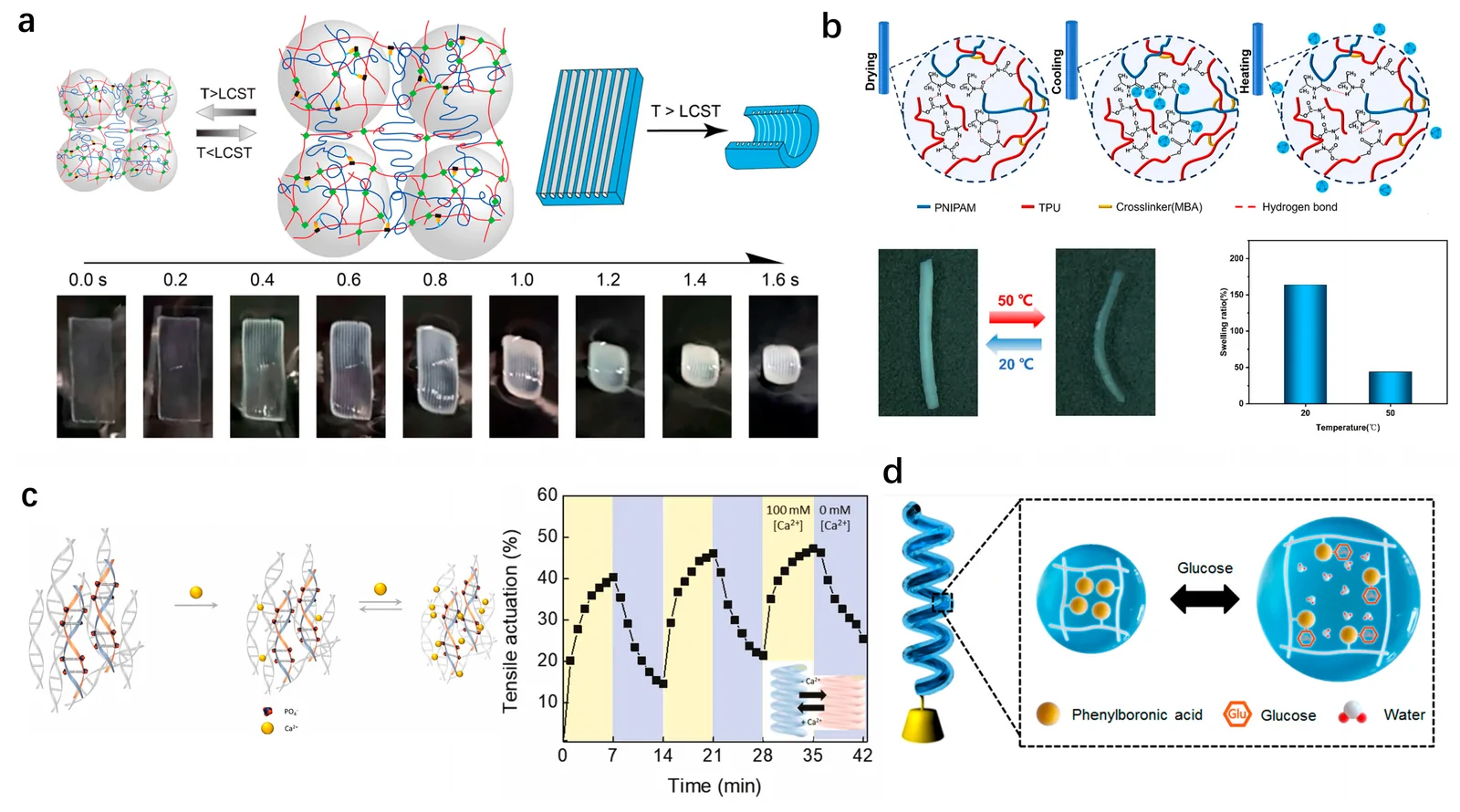
Thermoresponsive, ion-responsive, and biomolecule-responsive hydrogel fiber actuators. (**a**) Thermoresponsive contraction of P(NIPAM-HEMA)/PNIPAM semi-interpenetrating-network hydrogel microfibers enabled by LCST-induced dehydration and enhanced chain mobility (reprinted with permission from [61]). (**b**) Thermo-/water-responsive shape-memory actuation of PNIPAM–TPU composite hydrogel fibers governed by hydrogen bonding and polymer-chain entanglement (reprinted with permission from [67]). (**c**) Ca^2+^-driven contraction of DNA hydrogel artificial muscles and tensile actuation of coiled DNA hydrogel fibers (reprinted with permission from [74]). (**d**) Glucose-responsive tensile actuation of self-coiling core–sheath fibers based on reversible phenylboronic acid–glucose binding (reprinted with permission from [19]).

PNIPAM-based thermoresponsive hydrogel fibers can be strengthened through semi-interpenetrating networks and elastic polymer composites, which help address the intrinsic brittleness, slow response, and limited mechanical stability of conventional PNIPAM hydrogels. Liu et al. [61] constructed P(NIPAM-HEMA)/PNIPAM semi-interpenetrating-network hydrogels and fabricated core–sheath thermoresponsive hydrogel microfibers using a double-coaxial laminar-flow microfluidic device. In this system, the crosslinked PNIPAM network provided thermally induced dehydration and contraction near the LCST, whereas the linear P(NIPAM-HEMA) chains introduced a physical network through hydrogen bonding and chain entanglement, reducing the constraints imposed by covalent crosslinking and enhancing segmental relaxation (Figure 6a). Compared with conventional PNIPAM hydrogels, the semi-interpenetrating network exhibited faster dehydration-induced contraction and higher elasticity above 37 °C. When fabricated into core–sheath microfibers with a diameter of approximately 100 μm, the high surface-area-to-volume ratio further accelerated water diffusion and heat transfer. Bundles composed of multiple microfibers could rapidly contract and lift loads after heating above the LCST, indicating that the response rate of thermoresponsive hydrogel fibers is governed not only by the PNIPAM phase transition, but also by the network crosslinking mode, the mobility of linear polymer chains, and the mass-transport pathway determined by fiber dimensions.

Xie et al. [67] further prepared PNIPAM/TPU dual-responsive composite fibers through wet spinning and ultraviolet crosslinking (Figure 6b). In this system, PNIPAM served as the temperature- and water-responsive component, while thermoplastic polyurethane (TPU) acted as the reinforcing phase and shape-memory support network. NIPAM, MBA, photoinitiator, and different amounts of TPU were blended and then processed by wet spinning in a water coagulation bath, accompanied by ultraviolet curing to form composite fibers. PNIPAM swelled through water uptake below the LCST and collapsed through dehydration above the LCST, whereas the incorporation of TPU improved the mechanical integrity and shape-fixation ability of the fibers through hydrogen bonding and polymer-chain entanglement. The composite fiber containing 60% TPU exhibited the best overall performance, with a shape-fixity ratio of 86%, a shape-recovery ratio of 95%, and a water-responsive recovery ratio of approximately 88%. Compared with pure PNIPAM fibers, its tensile strength increased by approximately threefold and its elongation at break increased by approximately twofold.

LCST-based fibers offer reversible actuation with accessible chemistries, but thermal inertia and water transport limit frequency. The heating route is as important as the polymer transition: bulk heating is uniform but slow, whereas photothermal, Joule, or magnetothermal heating is local and fast but may create thermal gradients and safety constraints. Transition temperatures measured in pure water should not be assumed to remain unchanged in saline or biological media.

### 3.3. pH, Ion, and Biomolecule Responsiveness

pH-, ion-, and biomolecule-responsive hydrogel fibers typically contain ionizable groups or specific recognition motifs, such as carboxyl, amino, sulfonate, and boronic acid groups, as well as the phosphate backbone of DNA. Changes in external pH, ionic strength, or target-molecule concentration can alter the charge density, hydration state, Donnan osmotic pressure, and chain conformation within the hydrogel network, thereby inducing fiber swelling, contraction, or changes in modulus [19,74,83,84,85]. Compared with water/humidity- or temperature-responsive actuation, these mechanisms are more closely linked to physiological chemical signaling, making them particularly suitable for chemical artificial muscles, ion-responsive actuators, and biomolecule-recognition-based soft robotic units.

Early pH-responsive hydrogel fibers were mainly represented by hydrolyzed polyacrylonitrile (H-PAN)-based systems. Yu et al. [75] prepared H-PAN/gelatin composite hydrogel fibers through wet spinning, in which carboxylate groups generated by alkaline hydrolysis of PAN endowed the fibers with pH responsiveness. The ionization/deionization of these carboxylate groups under different pH conditions modulated electrostatic repulsion and osmotic pressure within the network, thereby driving reversible swelling or contraction of the fiber. The contraction of mammalian skeletal muscle is powered by ATP and regulated by ionic signals such as Ca^2+^. Inspired by this principle, Kim et al. [74] constructed Ca^2+^-driven DNA hydrogel fiber artificial muscles. The phosphate backbone of DNA provides a high density of anionic sites, and electrostatic coordination with Ca^2+^ changes interchain interactions and the contracted state of the network. DNA fibers obtained by wet spinning were first stabilized by Ca^2+^ treatment and then converted into twisted or coiled anisotropic structures through twisting or winding (Figure 6c). As the Ca^2+^ concentration increased, the DNA network contracted. In the twisted configuration, this contraction was converted into reversible rotation, with a maximum rotation angle of 183° cm^−1^. In the coiled configuration, it was transformed into axial contraction, with a maximum contraction ratio of 40.72% and a work capacity of 50.86 J kg^−1^. The key feature of this system is the coupling of ion-regulated network contraction with twisted/coiled geometry, which converts a Ca^2+^-regulated chemical process analogous to muscle signaling into reversible mechanical output.

Biomolecule-recognition responsiveness regulates the state of hydrogel networks through specific binding interactions. Sim et al. [19] designed glucose-responsive self-coiling core–sheath fibers using a pre-twisted nylon thread as the core and an in situ polymerized acrylamide hydrogel sheath containing phenylboronic acid groups (Figure 6d). After swelling in buffer solution, the hydrogel sheath exhibited a reduced modulus, disrupting the balance between the untwisting force of the nylon core and the restoring force of the sheath. As a result, the fiber spontaneously formed a reverse helical structure at room temperature. Upon reversible binding between glucose and phenylboronic acid, the hydrophilicity and swelling ratio of the sheath increased, while its modulus decreased. This allowed the untwisting force of the core to dominate, inducing reversible axial contraction. The self-coiling architecture amplified glucose-induced network swelling and modulus changes into a mechanical stroke, achieving a maximum tensile stroke of 2.3%, approximately six times that of non-coiled fibers, and a maximum work density of 130 kJ m^−3^. The helical core–sheath geometry amplified the swelling and modulus changes caused by glucose binding.

pH-, ion-, and biomolecule-responsive fibers provide chemical specificity that is unavailable to many field-driven actuators, but they are strongly affected by buffer capacity, ionic strength, competing ions, diffusion, and nonspecific adsorption. Reported equilibrium strokes should therefore be accompanied by measurements across concentration ranges and in media relevant to the intended application, together with selectivity and reversibility tests.

### 3.4. Electrical and Electromagnetic-Force Actuation

Electrical stimulation offers high temporal resolution and straightforward integration with control circuits, making it an important external field for closed-loop actuation in hydrogel-fiber-based soft robots. The main electrically driven mechanisms in hydrogel fibers include ion migration, electroosmotic flow, electrochemical reactions, conductive-network reconstruction, and direct electromagnetic-force actuation [12,72,73,86,87]. Compared with temperature- or humidity-responsive actuation, electrical stimulation more readily enables rapid switching and spatially selective control. However, its actuation efficiency depends strongly on ion-transport pathways, the distribution of conductive components, and how the electric field is applied within or around the fiber [3,18].

**Figure 7:**
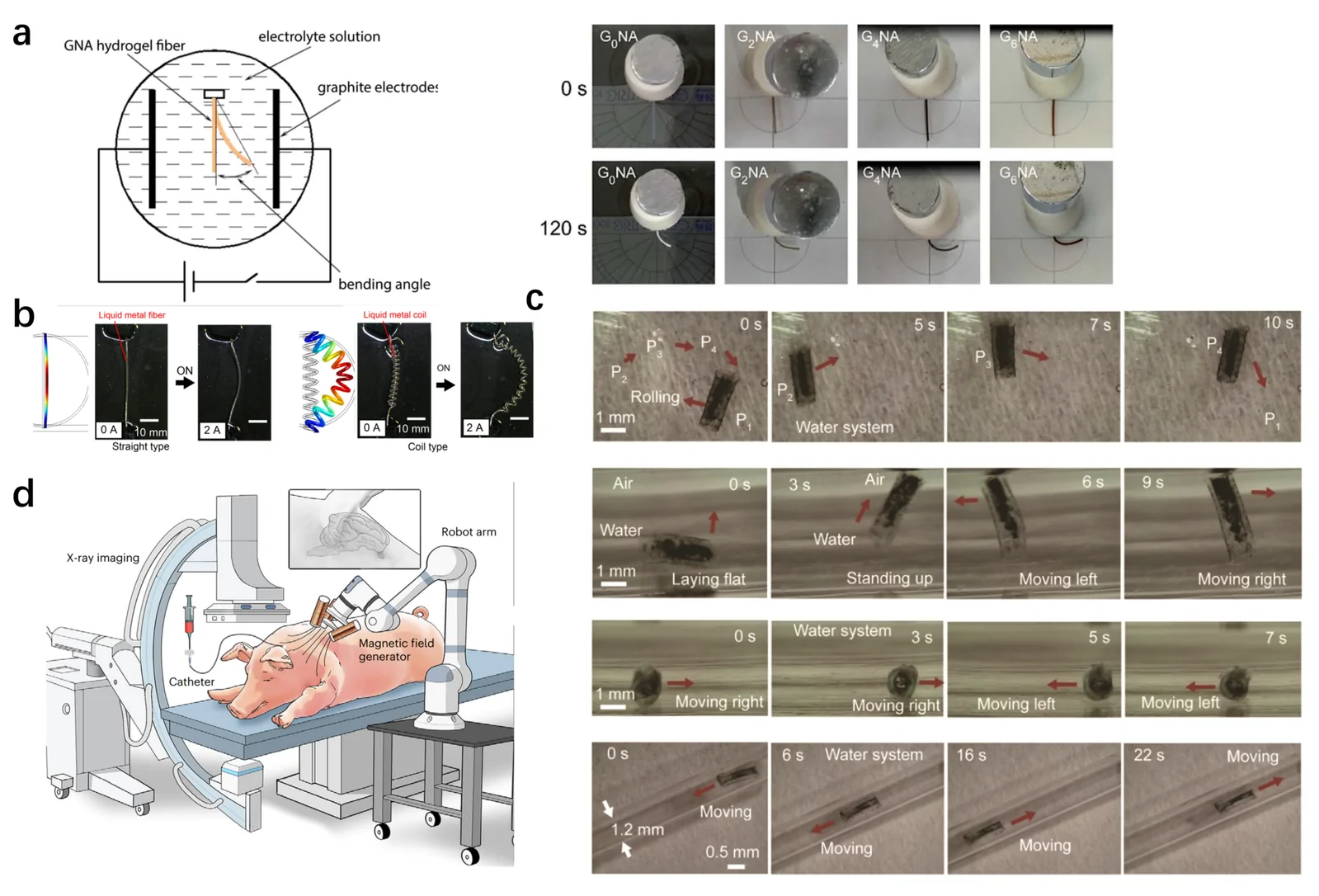
Field-driven hydrogel fiber actuation enabled by electrical, electromagnetic, and magnetic stimuli. (**a**) Experimental setup for electroresponsive bending of GO/PNIPAM/alginate hydrogel fibers driven by ion migration, osmotic-pressure gradients, and thermally induced contraction (reprinted with permission from [58]). (**b**) Straight and coiled liquid-metal hydrogel fibers for Lorentz-force-driven electromagnetic actuation under the combined application of an electric current and a magnetic field (reprinted with permission from [18]). (**c**) Magnetically steerable hollow hydrogel microrobots capable of rolling, jumping, and directional motion under external magnetic fields (reprinted with permission from [42]). (**d**) Fluoroscopy-guided magnetic delivery of biohybrid blood hydrogel fibers for targeted therapy in confined intracranial spaces (reprinted with permission from [37]).

Early electroresponsive hydrogel fibers mainly relied on ion migration within polyelectrolyte networks. For example, submicrometer chitosan hydrogel fibers prepared by electro-wet spinning exhibited ion-responsive behavior in the swollen state. An external electric field induced ion redistribution within the fibers and generated local osmotic-pressure gradients, thereby producing reversible electrically induced contraction or bending [10]. Graphene oxide (GO)-based composite hydrogel fibers represent an important strategy for improving electroresponsive speed and mechanical stability. Peng et al. [58] incorporated GO into a PNIPAM/alginate network to construct GO/PNIPAM/SA hydrogel fibers with both thermoresponsive and electroresponsive properties. These fibers were fabricated through microfluidic spinning, Ca^2+^ crosslinking of the alginate template, and subsequent PNIPAM polymerization, followed by removal of Ca^2+^ to obtain a semi-interpenetrating network composed of crosslinked PNIPAM and linear alginate. The electrical response of this system was still mainly driven by osmotic-pressure gradients generated by anionic groups on alginate and GO. However, unlike simple PAAm/alginate systems, the incorporation of PNIPAM introduced thermal phase-transition regulation, causing the fibers to undergo both ion-migration-induced bending and Joule-heating-induced contraction under an electric field. At the initial stage of direct-current stimulation, the fiber bent toward the cathode owing to the osmotic-pressure difference. As the electrolyte temperature increased and approached the volume phase-transition temperature of PNIPAM, the PNIPAM network dehydrated and contracted, leading to reverse bending and axial shortening of the fiber. Increasing the GO content further enhanced electroresponsive bending because the oxygen-containing functional groups increased the polyelectrolyte density and strengthened the local electric-field effect through polarization (Figure 7a). The observed motion therefore combines ion-migration-induced bending with Joule-heating-induced PNIPAM contraction in electroresponsive hydrogel fibers, providing a materials basis for designing multistimuli-responsive artificial muscles.

In addition to ion migration and osmotic-pressure-gradient-driven deformation, electric fields can also induce sustained oscillations in hydrogel fibers through fluid–structure-coupled instabilities. Boiardi et al. [88] investigated the flutter instability of polyelectrolyte gel filaments under a steady and uniform electric field, and introduced natural curvature into the filaments during fabrication to break left–right symmetry. This system differs from conventional reciprocating bending driven by alternating electric fields. Instead, it uses the coupling among steady and uniform electrical stimulation, morphoelastic filaments, and viscous fluids to generate nonreciprocal oscillations with a net thrust once a critical condition is reached. Because such oscillations are nonreciprocal under low-Reynolds-number hydrodynamics, the filaments can generate net thrust on the surrounding fluid. The natural curvature further regulates the thrust direction, making it possible for artificial cilia arrays to generate spatially programmable flow fields under a uniform constant electric field. This work provides a mechanistic route for electrically driven hydrogel fibers that is distinct from osmotic bending: through natural curvature and flutter instability, a simple steady and uniform electric field can be converted into self-sustained, nonreciprocal periodic motion with net thrust.

Tachibana et al. [18] proposed a more direct electromagnetic actuation mechanism. They fabricated alginate/PVA hydrogel microtubes by microfluidics and introduced a liquid-metal conductive core into the lumen, forming soft liquid-metal hydrogel fibers. When the fiber was placed in an external static magnetic field and driven by alternating or direct current, the current flowing through the liquid metal interacted with the magnetic field to generate Lorentz forces, directly pulling and deforming the surrounding hydrogel network. This mechanism bypasses the rate limitations associated with water diffusion and ion migration, enabling a rapid response speed of 260.5 mm s^−1^, local high-frequency control at 6 Hz, and large deformation of 172%. In this system, the liquid metal provides a low-resistance conductive pathway, the hydrogel shell serves as a soft deformable matrix, and the external magnetic field converts electrical input into an immediate mechanical force (Figure 7b). Compared with conventional electroresponsive hydrogel fibers, this strategy shifts the actuation source from mass transport within the polymer network to direct field-force coupling, making it particularly suitable for high-frequency, rapid, and locally controllable soft actuation.

Electrical inputs provide high temporal programmability, yet high voltage or current can introduce Joule heating, electrolysis, gas formation, and local pH gradients that become unintended actuation pathways. Direct Lorentz-force coupling can bypass diffusion and achieve rapid motion, but it requires conductive encapsulation, substantial current, and an external magnetic field. Electrical performance should therefore be separated into ionic, electrochemical, thermal, and direct force contributions.

### 3.5. Magnetic Responsiveness

Magnetic-field stimulation offers non-contact actuation, strong penetration, remote controllability, and spatial localization, making it particularly suitable for untethered soft robots, in vivo microactuators, and targeted delivery systems [37,42]. Magnetic hydrogel fibers generally operate through two main routes. The first involves incorporating magnetic particles, such as Fe_3_O_4_ nanoparticles or carbonyl iron particles, into the hydrogel network, so that external magnetic fields can generate forces or torques to drive fiber bending, rolling, swinging, or crawling. The second uses magnetic fields during fabrication to align or asymmetrically distribute magnetic components, thereby creating programmable anisotropic architectures. In this context, magnetic components act not only as actuation units, but also as functional elements for structural programming, imaging, tracking, and quality inspection [30,89,90].

Liang et al. [42] combined magnetic responsiveness with hollow hydrogel microfibers to fabricate magnetically steerable tubular microrobots. They used a non-coaxial microfluidic method, in which solid sodium alginate microfibers were first extruded and then introduced into a Tris–HCl buffer containing CuSO_4_ and H_2_O_2_. Cu^2+^ crosslinked the carboxyl groups of alginates to fix the fiber morphology, while simultaneously catalyzing the decomposition of H_2_O_2_ to generate oxygen bubbles, thereby transforming the solid fibers into continuous hollow structures. By adjusting the nozzle number, the distance between the nozzle and the liquid surface, and the flow rate, single-helical spring, double-helical spring, necklace-like, and ladder-like hollow microfibers could be obtained. After loading magnetic particles, these hollow microtubes could perform rolling, jumping, and directional motion inside capillaries under an external magnetic field of 100–120 mT, and could push obstructions in simulated vascular channels (Figure 7c). This system integrates hollow lumens, complex geometry, and magnetic navigation, highlighting the structural advantages of magnetically responsive hydrogel fibers for microscale tubular robots.

Wang et al. [37] constructed biohybrid blood hydrogel fibers using autologous blood to reduce the risk of immune recognition and macrophage clearance associated with exogenous materials. In this system, low-dose magnetic nanoparticles were mixed with a small amount of fresh blood, and the coagulation cascade was used to form magnetic blood hydrogel microfibers within approximately 15 min. The blood-gel network provided a biologically derived matrix, while the magnetic particles enabled external magnetic manipulation and real-time in vivo tracking under X-ray fluoroscopy. These microfibers could move through narrow cerebrospinal fluid spaces and release drugs on demand after reaching the target region (Figure 7d). By integrating biological origin, magnetic actuation, imaging visibility, and drug delivery within a single hydrogel fiber, this system offers a potential route toward personalized in vivo soft microrobots.

Magnetic actuation offers remote penetration and does not require a wired connection to the fiber, but direct magnetic force/torque and magnetothermal heating must be distinguished. Particle concentration increases magnetic output while also changing modulus, density, optical loss, heating, and aggregation. Field gradient, flux density, frequency, particle magnetization, and medium viscosity should be reported to make magnetic results comparable.

### 3.6. Cross-Mechanism Comparison and Design Selection

Actuation mechanism and fiber architecture should be selected together, although the available energy source and required response time usually provide the first constraints. Humidity-, temperature-, pH-, ion-, and biomolecule-responsive systems can generate large strains and interact directly with chemical environments, but their response is generally limited by mass transport. Magnetic actuation enables remote control through opaque media, whereas photothermal actuation offers localized stimulation but requires control of heat transfer and irradiation geometry. Electrical and electrochemical mechanisms are readily integrated with electronic control but may introduce tethering, Joule heating, and electrode reactions. Direct electromagnetic-force actuation can respond more rapidly than diffusion-controlled systems, although it requires conductive encapsulation, substantial current, and magnetic hardware.

Architecture determines how the resulting strain or force is converted into motion. Janus and bilayer fibers favor bending, twisted structures convert strain into torsion or axial contraction, hollow fibers add pumping and transport functions, and core–sheath or printed structures facilitate functional integration. Design selection should therefore consider the robotic task, required degree of freedom, energy input, mechanical load, environmental conditions, and manufacturing constraints together.

Free stroke alone is insufficient for comparing robotic performance. Blocked force or stress, work density, response and recovery times, energy consumption, fatigue life, environmental stability, and device-level performance should also be reported under clearly defined hydration and loading conditions.

## 4. Challenges, Translation, and Future Perspectives

Most hydrogel-fiber soft robots are still evaluated as isolated components under controlled laboratory conditions. Practical use requires stable performance after storage, assembly, cyclic loading, environmental variation, and integration with sensing, power, packaging, and control systems. The following sections discuss the main barriers related to material lifetime, secondary stimuli, actuation speed, signal reliability, autonomy, manufacturing, and translation.

### 4.1. Long-Term Stability and Lifecycle Reliability

The compliance, stretchability, ionic conductivity, and biocompatibility of hydrogel fibers depend strongly on their hydrated polymer networks. When exposed to open air for prolonged periods, water loss can restrict polymer-chain mobility, weaken ion transport, and compromise mechanical performance. At low temperatures, ice-crystal formation may further disrupt the network structure, leading to irreversible damage. As a result, dehydration and freezing remain major barriers to the use of hydrogel-fiber-based soft robots outside wet laboratory environments, particularly in air-exposed, low-temperature, or otherwise complex outdoor settings.

Anti-dehydration approaches include elastomer encapsulation, hydrophobic or oil-based barriers, organohydrogel solvents, hygroscopic components, poly(ionic liquids), dense hydrogen-bonding networks, and dynamically crosslinked matrices [91]. Each strategy creates a trade-off: barriers may suppress mass exchange and slow chemical actuation, while salts and organic cosolvents can shift phase transitions, change conductivity, or compromise biocompatibility. Reliability tests should therefore reproduce the intended service medium and load. At minimum, studies should report changes in mass and diameter, retention of modulus and conductivity, blocked force or stroke retention, creep under sustained load, interfacial integrity, and particle leakage after storage and cycling in air, controlled humidity, deionized water, saline, buffer, serum-containing medium, or other application-specific environments.

For wearable or industrial systems, packaging, shelf life, freeze–thaw resistance, and performance after repeated drying and rehydration are decisive. For implanted or intraluminal systems, sterilization, protein adsorption, biofouling, enzymatic degradation, immune response, and retrieval or biodegradation must be considered. A credible durability claim should include both accelerated aging and realistic use conditions and should distinguish reversible baseline drift from irreversible network damage.

### 4.2. Secondary Stimuli and Environmental Cross-Coupling

Stimuli-responsive hydrogel fibers rarely respond to a single independent variable. Their state is a multivariate function of temperature, pH, ionic strength, buffer identity, humidity, solvent composition, mechanical load, light absorption, field history, and exposure time. A variable that is secondary to the intended input can shift the response threshold, alter swelling, or even reverse the deformation direction. This cross-coupling should be distinguished from deliberately designed multi-stimulus behavior: the former is an error source, whereas the latter is a controlled function.

Shymborska et al. showed that buffer composition and pH substantially changed the LCST, wettability, and morphology of poly(oligo(ethylene glycol) methyl ether methacrylate) (POEGMA) brush coatings because buffer ions penetrated the polymer layer and disrupted its hydration shell [92]. Although that work concerns grafted coatings rather than fibers, the principle is directly relevant to thermoresponsive hydrogel-fiber systems: phase transitions measured in pure water cannot be transferred uncritically to phosphate-buffered saline, culture medium, or physiological fluid. Similar coupling occurs when electric fields generate Joule heat and electrode-induced pH gradients, when near-infrared light simultaneously heats and dehydrates a fiber, or when magnetic nanoparticles generate both force and heat [18].

A robust characterization protocol should vary the intended stimulus and the most plausible secondary variable independently, then test their interaction using a factorial or response-surface design. Controls should include matched temperature, pH, ionic strength, and solvent conditions; real-time thermal, electrical, and mass measurements; and baseline correction after equilibration. Table 4 summarizes common cross-couplings and the minimum controls needed to identify them.

### 4.3. Breaking the Speed Limit of Diffusion-Controlled Actuation

Many hydrogel fiber actuators rely on the transport of water, ions, or solvents within polymer networks. LCST-based thermoresponsive networks, pH-responsive networks, and osmotic-pressure-driven systems are therefore intrinsically limited by diffusion kinetics. According to Fickian diffusion, the response time generally scales with the square of the characteristic length. Fabricating hydrogels into microscale fibers, introducing hollow lumens, or increasing the specific surface area can shorten radial mass-transport distances. However, for macroscale, high-frequency, and continuous robotic motion, reducing structural dimensions alone remains insufficient. At present, the operating frequencies of many diffusion-limited hydrogel actuators are still far below those required for biological high-frequency motions, such as insect wing flapping, ciliary beating, or cardiac contraction.

Future efforts to improve response speed should reduce the dependence of actuation on mass transport and develop non-diffusion or weakly diffusion-dependent mechanisms. One route is direct field-force actuation. For instance, liquid-metal hydrogel fibers can generate Lorentz forces through the coupling between an external magnetic field and electrical current, thereby bypassing the rate limitation associated with water transport into and out of the network and enabling rapid, large-deformation responses [18]. Another route is to exploit fluid–structure-coupled instability under a constant electric field, allowing fibers to generate self-sustained oscillations in fluid rather than relying on continuous modulation by alternating stimuli. In addition, photoisomerizable molecules may be introduced so that light directly triggers chain-conformation changes, thereby shortening the timescale from energy input to mechanical output. In future high-frequency hydrogel fiber actuators, the design focus should further shift from “accelerating water diffusion” to “reducing the dependence of actuation on diffusion”.

### 4.4. Eliminating Multimodal Signal Crosstalk and Nonlinear Errors

Closed-loop soft robots require not only actuation capability, but also accurate perception of their own deformation and external contact states. However, hydrogel sensing networks often exhibit pronounced viscoelasticity and sensitivity to multiple stimuli. During dynamic cyclic stretching, energy dissipation within polymer networks can cause hysteresis, stress relaxation, and electrical signal drift. At the same time, piezoresistive, capacitive, and ionically conductive sensors may respond simultaneously to strain, temperature, humidity, and ionic strength. When different stimuli produce similar signal changes, the control system may struggle to distinguish true contact or structural deformation from environmental temperature or humidity disturbances.

Solving signal crosstalk cannot rely solely on a single material formulation. A more feasible route is to combine front-end structural decoupling with back-end algorithmic calibration. At the front-end design level, microfluidics or micro/nanoscale 3D printing can be used to construct star-shaped, semicircular, flat rectangular, or gear-shaped cross-sections, enabling fibers to generate direction-dependent responses to different strain modes and thereby structurally distinguish mechanical signals from environmental perturbations [93]. Hydrogel optical-fiber or grating-based sensing networks can reduce errors caused by electromagnetic interference and conductive-liquid short circuits, making them suitable for soft sensing in magnetic resonance imaging (MRI), strong electric fields, or body-fluid environments. At the back-end processing level, machine learning and long short-term memory (LSTM) networks can be used to compensate for hysteresis, baseline drift, and environmental cross-responses [94]. For complex tasks such as blind grasping by soft manipulators, positioning of miniature medical catheters, and in vivo motion feedback, material structure, sensing architecture, and data models must be designed in a coordinated manner.

### 4.5. Untethered and Self-Powered Integrated Soft Systems

Untethered hydrogel-fiber robots require compact sources of energy and control. Fiber arrays may combine actuation with ionic, optical, or chemical signal transmission, but most reported systems still rely on external light sources, magnetic-field generators, electrodes, or pumps [29]. Enzymatic fuel cells and other soft energy-harvesting components could provide local power in future systems, although their output, lifetime, and compatibility with hydrated actuators remain insufficiently established. For biomedical use, integration with blood or host tissue must also be evaluated in terms of inflammation, fouling, degradation, retrieval, and long-term functional stability [37]. Progress toward autonomy will therefore depend on reducing power demand and integrating actuation, sensing, and control without substantially increasing stiffness or device size.

### 4.6. Technology Readiness, Commercialization, and Clinical Translation

Most hydrogel-fiber soft robots remain at the material or component proof-of-concept stage. Reported studies commonly use short fibers, controlled liquid environments, and benchtop light, electrical, or magnetic equipment, whereas production yield, shelf life, packaging, sterilization, and cost are rarely reported. Continuously spun and knittable fibers are more compatible with textile manufacturing than precision microfluidic fibers, but their device-level reliability under storage, washing, drying, and repeated loading still requires validation [51].

Translation requirements differ by application. Adaptive textiles and environmental devices may tolerate relatively slow responses and external humidity or light sources, provided that dehydration, washability, and connection durability are controlled. Implantable fibers and biomedical microrobots face additional requirements, including sterile manufacture, hemocompatibility, particle retention, biofouling, imaging, navigation, dose control, degradation or retrieval, and validation in relevant animal models [37,95]. Locomotion in a simplified laboratory channel should therefore be described as proof of concept rather than evidence of clinical readiness.

Commercial evaluation should emphasize process capability as well as peak actuation performance. Relevant metrics include batch variation, continuous run length, defect rate, packaging yield, storage stability, energy consumption, sterilization resistance, and device-level failure modes. Inline monitoring of fiber diameter, conductivity, optical attenuation, or particle distribution may help establish reproducible manufacturing specifications.

## 5. Conclusions

Hydrogel fibers combine material responsiveness with cross-sectional and axial anisotropy, allowing for swelling, phase transitions, ion transport, and field-induced forces to generate directional motion. Janus and bilayer structures are effective for bending; core–sheath structures separate and protect functional domains; hollow fibers couple actuation with fluid transport; twisted structures amplify strain into rotation or contraction; and patterned, woven, or printed fibers enable more complex motion at the device level. Architecture should therefore be selected according to the required deformation, load, transport function, and manufacturing route.

Nanofillers can improve mechanical reinforcement or introduce electrical, magnetic, photothermal, and catalytic functions. Their benefits depend on dispersion, orientation, connectivity, and interfacial stability rather than filler content alone. Across the different actuation mechanisms, the main trade-offs are between response speed and transport distance, force and compliance, multifunctionality and structural complexity, and environmental sensitivity and actuation amplitude.

Future studies should report blocked force, work density, response and recovery times, fatigue life, and environmental stability under clearly defined testing conditions. Continuous manufacturing, stable interfaces, control of secondary stimuli, packaging, and closed-loop operation remain necessary for translating hydrogel-fiber actuators from laboratory demonstrations into reliable soft robotic devices.

## Figures and Tables

**Table 1:** Comparison of fabrication strategies for anisotropic hydrogel fibers.

Fabrication Method	Main Advantages	Main Limitations	Best Suited for
Wet spinning	Continuous and long-fiber production	Limited structural precision	Fiber actuators and textile robots
Continuous photopolymerization	Rapid continuous forming	Sensitive to oxygen and light penetration	Knittable and spoolable fibers
Microfluidic spinning	Precise control of complex structures	Low throughput and possible clogging	Janus, core–sheath, and hollow fibers
Electrospinning	High surface area and fiber alignment	Difficult to obtain individual hydrated fibers	Fibrous mats and membrane actuators
Template method	Simple and widely applicable	Batch fabrication and poor scalability	Laboratory-scale fiber preparation
Multimaterial extrusion printing	Programmable geometry and direct integration	Slow printing and strict ink requirements	Complex soft robotic structures

**Table 2:** Comparative design characteristics of anisotropic hydrogel-fiber architectures for soft robotics.

Architecture	Actuation Principle	Main Advantage	Main Limitation	Suitable Applications
Janus/bilayer	Strain mismatch between two sides	Simple structure and directional bending	Possible interfacial delamination; mainly single-plane motion	Grippers, valves, and bending actuators
Core–sheath	Radial confinement and functional separation	Good structural stability and multifunctional integration	Complex coaxial fabrication; sheath may slow mass transport	Artificial muscles and optical/electrical fibers
Hollow	Lumen-volume change and thin-wall deformation	Fast mass transport and internal loading space	Lumen collapse, leakage, and low compressive strength	Micropumps, delivery systems, and tubular robots
Helical/twisted	Conversion of swelling into torsion or axial motion	Large rotational or contractile deformation	Sensitive to twist geometry, creep, and fatigue	Rotary actuators, yarn muscles, and smart textiles
Gradient/patterned	Spatially programmed responsive strain	Programmable local and multistep deformation	Difficult fabrication control and limited pattern resolution	Tendril-like actuators and programmable folding
Woven/printed network	Coordinated deformation of multiple fibers	Complex, multi-axis, and device-level motion	Complicated assembly and uneven load distribution	Locomotion robots, manipulators, and adaptive textiles

**Table 4:** Primary stimuli, secondary environmental variables, and recommended controls for hydrogel-fiber actuators.

Primary Stimulus	Important Secondary Variables	Possible Interference	Recommended Controls
Water/humidity	Temperature, airflow, ionic strength	Changes in swelling rate and baseline deformation	Control humidity and temperature; equilibrate samples before testing
Temperature	Heating rate, hydration state, medium composition	Uneven heating, dehydration, and thermal drift	Use temperature feedback and fixed heating conditions
Light/photothermal	Light intensity, irradiation distance, ambient temperature	Nonuniform heating and water loss	Calibrate optical power; monitor fiber temperature
pH/ions/biomolecules	Buffer composition, ionic strength, competing species	Nonspecific swelling and cross-sensitivity	Use matched control solutions and concentration calibration
Electrical field	Electrolyte conductivity, electrode polarization, Joule heating	Coupled ionic and thermal responses	Standardize electrolyte conditions; monitor current and temperature
Magnetic field	Field strength/gradient, particle loading, fluid viscosity	Variation in magnetic force, torque, and motion resistance	Calibrate the magnetic field and standardize particle content

## Data Availability

No new data were created or analyzed in this study.
